# Rapid assessment of the factors contributing to the increase in maternal mortality during the COVID-19 pandemic in the Latin American region

**DOI:** 10.1186/s12884-025-08069-y

**Published:** 2026-01-03

**Authors:** Evelina Chapman, Silvina Ramos, Mariana Romero, Guido Sciurano, Jim Ricca, Gloria Metcalfe, Jovita Ortiz Contreras, Joaquín Gómez Dávila, Daniel Camilo Aguirre Acevedo, Jorge Hermida Cordova, Alma Virginia Camacho-Hubner

**Affiliations:** 1 Center for the Study of State and Society, CEDES, Buenos Aires, Argentina; 2 Department of Public Health, University of the Frontier, Temuco, Chile; 3 CONICET - Center for the Study of State and Society, CEDES, Buenos Aires, Argentina; 4 Johns Hopkins Bloomberg School of Public Health, Baltimore, MD USA; 5 Jhpiego, MNH Consultant, Baltimore, USA; 6 Women’s and Newborn Health Promotion Department, Universidad de Chile, Santiago, Chile; 7 NACER Group, University of Antioquia, Medellín, Colombia; 8 Foundation for Health Services Research and Management, FIGESS, Quito, Ecuador; 9 UNFPA Latin America and the Caribbean Regional Office, Regional Adviser in Sexual and Reproductive Health, Clayton, Panama

**Keywords:** COVID-19, Three delays model, Implementation, Health policy, Maternal mortality, Maternal health, Reproductive health

## Abstract

**Background:**

COVID-19 infection in pregnant women was known to be associated with increased morbidity and mortality in Latin America and the Caribbean as a consequence of comorbidity and disruption in the supply and use of health services.

**Methods:**

A multi-country qualitative study was carried out in Chile, Colombia, and Ecuador to investigate the factors contributing to maternal mortality in the period March 2020 - July 2021. Four sources were analyzed: health policy documents and interviews with decision-makers, service providers of health and relatives of women who died due to maternal causes during the aforementioned period. The information collected was coded according to dimensions of the SURE Collaborative model (Supporting the Use of Research Evidence Collaborative) for the analysis of the implementation of health policies; and their implementation was analyzed by applying the Three Delays model. Sixty-two policy documents were analyzed, and 21 interviews with decision makers, 30 interviews with service providers and 28 interviews with relatives of women who died from maternal causes were conducted.

**Results:**

The most relevant findings were the change in the maternal and reproductive health care model with the disruption of primary health care; the prioritization of emergency care for patients affected by COVID-19; and the fear of pregnant women to seek health services. The atomization of health management and the problems of communication/dissemination of the measures aimed at the general population and health teams generally undermined the provision of quality maternal and reproductive health services.This was exacerbated by socioeconomic vulnerability and lack of systematic policy implementation, as exemplified by the uneven rollout of telemedicine and home visits. Resource and skill gaps affected both the healthcare system and service users, particularly impacting the third delay in the maternal and reproductive health continuum. Deficiencies in infrastructure, supplies, human resources, and their protection further compounded these challenges.

**Conclusion:**

Various factors affected the availability, use, and quality of maternal, and reproductive health services during the COVID 19 pandemic. Access to timely quality maternal health care was severely affected.

**Study registration:**

The study protocol was registered on the OSF storage website (Chapman et al. 2022. 10.17605/OSF.IO/36JQD).

**Supplementary Information:**

The online version contains supplementary material available at 10.1186/s12884-025-08069-y.

## Background

The COVID-19 pandemic had negative effects on the health of pregnant women and numerous studies which pointed toward an increase in the Maternal Mortality Ratio (MMR), ranging from 8.5 to 65.5% [2]. This evidence was from early in the pandemic [3–5]. A study carried out in eight Latin American countries [6] showed that approximately a third of women who died of maternal causes had not been admitted to the intensive care unit nor had they received respiratory care, which was interpreted by the authors as indicating the existence of barriers to access due to the limited number of beds, problems with the referral processes, the overburdening of the health systems and the lack of critical care beds in these countries. Although studies identify the increase in maternal mortality with COVID-19, in addition to reports of excess mortality among non-pregnant women with COVID-19 and among pregnant women without COVID-19, reports in the LAC region of excess population mortality explained by this disease are scarce.

Analysis of vital statistics data from Chile [7], Colombia [8] and Ecuador [9] focusing on maternal mortality during the pandemic compared to a baseline of expected deaths generated from data from the previous five years (2015 a 2019), showed that there was a significant increase in the number of maternal deaths. This increase was 161% for Chile, 41% for Colombia and 46% for Ecuador in 2020 (see supplementary files). In all three countries, the increases in maternal deaths were due to increases in both direct and indirect obstetric causes, the latter being higher. Compared to 2019, deaths due to direct obstetric causes increased by 55% in Chile, 30% in Colombia and 43% in Ecuador, while deaths due to indirect obstetric causes increased 340%, 62% and 75% respectively (see supplementary files).

Another study from Brazil [10], based on twenty-five in-depth interviews with relatives of women who died due to maternal causes during the pandemic, identified three barriers to access to care, referring to the three delays: difficulty in identifying symptoms of COVID-19, delays in hospitalization attributed to the reluctance of health personnel to admit pregnant women, and delays in admission to intensive care units.

With this regional context, we carried out a qualitative study in Chile, Colombia, and Ecuador with the objective of analyzing the maternal and reproductive health policies formulated and implemented during the COVID-19 pandemic from the perspective of decision-makers, health service providers and family members of women who died from maternal causes and identified barriers and facilitators of the behaviors needed to avoid those deaths.

## Methods

This is an assessment based on qualitative survey techniques and analysis of primary information [11], carried out in Chile, Colombia, and Ecuador. The research team was composed of a supervision team from UNFPA and the MOMENTUM Country and Global Leadership project; a general coordinating team made up of CEDES researchers and a local research team in each country, who were responsible for collecting and analyzing the information.

The study was implemented simultaneously in the three countries in two stages. The first included the analysis of documents on health policies, strategies and guidelines issued by the highest national health authority related to the management of the pandemic and maternal, perinatal, and reproductive health care, published between March 2020 and July 2021. The second stage consisted of semi-structured interviews with decisionmakers, providers and relatives of women who died from maternal causes to reveal their perspectives on the implementation of these policies and the factors that could act as barriers or facilitators to care.

For the documentary analysis, all the documents of policies, strategies and guidelines were included. Policies examined were both those that were general in scope for the COVID-19 pandemic and those that specifically referred to access to and care for maternal, perinatal, and reproductive health, published between March 2020 and July 2021.

For the interviews with key informants, purposive samples were obtained in each of the three countries, consisting of:

- Seven health decisionmakers, according to the administrative configuration of the national health systems and with key management functions in the management of health policy at different administrative levels (national and subnational).
- Ten service providers from different levels of maternal, perinatal, or reproductive health care from different regions of the country and professional profiles (midwifery nurse, midwives/midwives/midwives, doctors or obstetricians and general practitioners).
- For the interviews with relatives, a sample of 20 women who died in each country due to causes related to pregnancy, childbirth or the puerperium was selected, stratified by diagnosis of confirmed or suspected COVID-19 and not associated with COVID-19, whose cause data of death and contact details of their relatives were available in the ordinary public records of each country. To capture the diversity of social and cultural conditions that could have affected access or quality of care, other variables were also considered, such as the place of residence of the deceased woman (urban or rural), age, ethnicity or race, immigration status, place of death (institutional or non-institutional), socioeconomic status. The relatives or people close to the deceased who answered the interview had to be over 18 years of age.

The period of action of decision-makers, providers and the occurrence of maternal deaths was the same as that considered for the policy documents. The search strategies and sources of information and the conformation of the samples of interviewees are detailed in the supplementary files.

The documentary analysis instrument was prepared using the implementation framework for policies proposed by the SURE Collaborative (Supporting the Use of Research Evidence Collaborative) adapted [12] to order the elements of the policies, the health system, and the local context. Domains were adapted to help identify potential barriers and facilitators at different levels, both at the level of the structure and the processes of the health system, for example, mechanisms of diffusion or dissemination of policies to health teams or to the public; availability or expansion of services; mechanisms to facilitate the accessibility/use of services; management or leadership; availability of information systems and updated care standards, among others [12].

For the preparation of the semi-structured interview guides for use with decision-makers and providers, in addition to the SURE model, the model of the three delays was considered [13, 14]. In this model, the first delay is defined as the recognition of the problem and the decision-making process to seek care. In general, it is multifactorial, but predominantly related to factors of the woman and her environment. The second delay refers to accessing health service. The third delay refers to obtaining timely and quality care.

The question guide of the instruments for decision-makers and providers covered dimensions such as the sociodemographic profile, perceptions regarding the pandemic and its impact, perceptions of the health policies or strategies implemented and their effects on maternal health, maternal and perinatal death, the deficits, and possible prospective adjustments. Health providers were also asked about the specific effects in their field of work.

The questionnaire for relatives of deceased women covered dimensions such as socioeconomic level of the deceased woman and her family; race/ethnicity; migrant status; conditions of access and use of the health system; relevant preconception and prenatal history; comorbidities; data on pregnancy potentially linked to death, including confirmed or suspected COVID-19, and other data relevant to maternal and perinatal death. The question guide for this instrument was designed ad hoc based on instruments used by the maternal mortality surveillance and prevention committees and previous studies [15]. The objective was the reconstruction of the trajectories that led to the death of women due to maternal causes [16], seeking to sequentially demonstrate the barriers and facilitators of access to health services and the quality of care received [17].

In the case of decision makers and providers, the interviews were conducted by telephone or through online platforms. In the case of relatives of the deceased women, most were through face-to-face meetings, and only four were carried out electronically. In all cases consent was obtained for the recording.

Both for the documentary analysis and for the interviews, data collection instruments were developed with instructions and local researchers were trained for their application, as well as for the review and analysis of information. All the instruments were locally validated before their application.

All four instruments can be found in the supplementary files.

The documentary analysis included the review of the corpus of documents using the policy implementation elements proposed by the SURE Collaborative model, in order to identify potential barriers and facilitators that could have existed when implementing the formulated policies.

The national research teams were in charge of the search, selection, data loading and analysis of the documents. The coordinating team developed a common matrix for data loading, supervised the decision to include documents, and cross-checked the searches and contents according to the SURE model. The documents were analyzed for evidence of systematic planning for implementation and the potential barriers that could have occurred to respond to the needs of women during pregnancy, childbirth and the postpartum period, including for emergencies.

The interviews were transcribed, and content analysis was carried out based on the model of the three delays [13, 14], as well as the availability, access, utilization, and quality of health services, to account for the perception of the interviewees on the implementation of the policies.

The data were coded in matrices and compared, looking for relationships and patterns that were significant to understand the circumstances of the deaths from the point of view of the relatives, with particular emphasis on those factors that could have hindered the access to and utilization of quality health services [13].

## Results

**Fig. 1:**
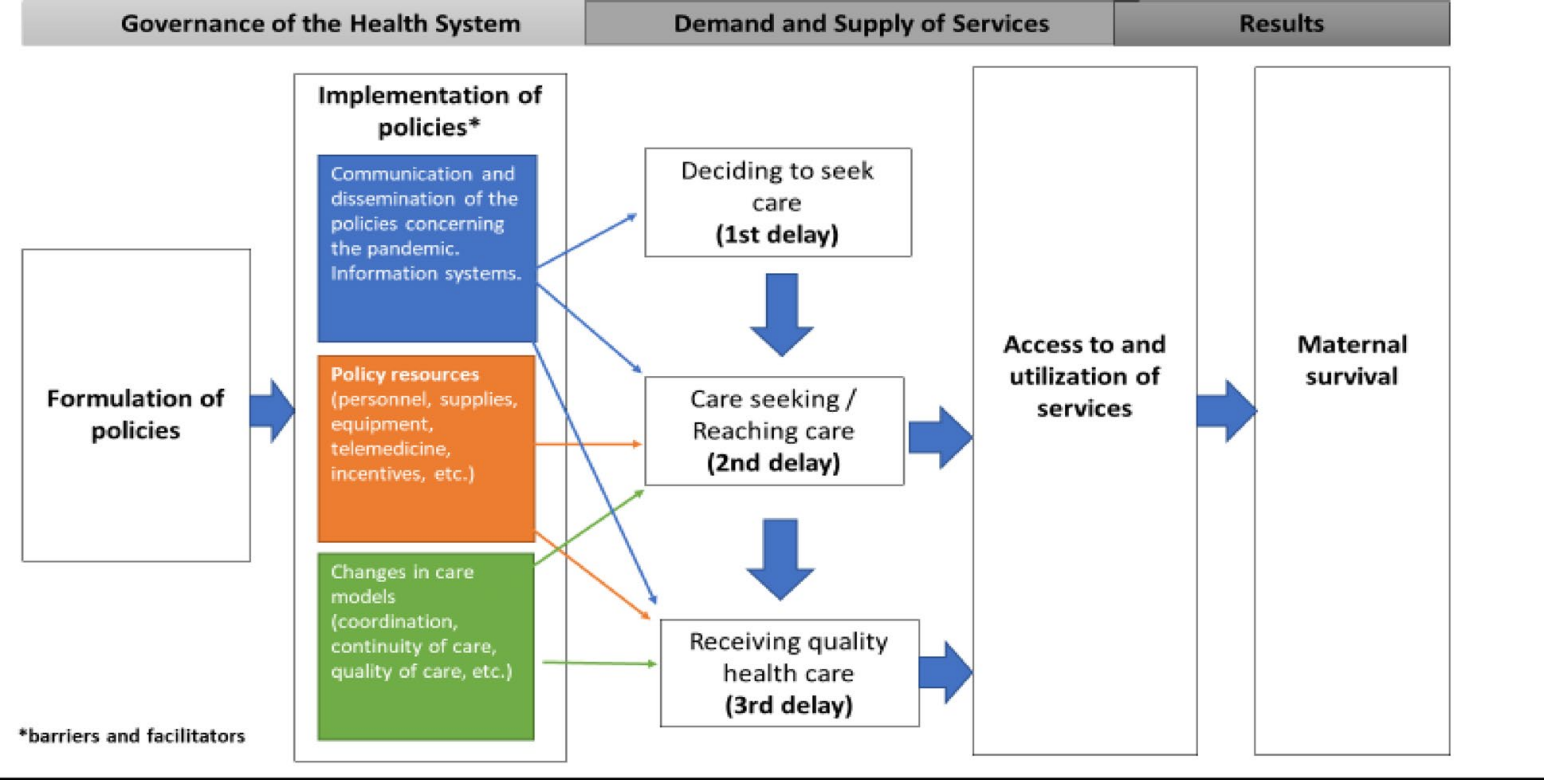
Logic model for the analysis, integrating the SURE and three delays models

The results of the analysis of the policies (document review) and their implementation from the perspective of the different actors interviewed are presented in an integrated manner and do not identify the country of origin. Figure 1 summarizes the logic model used for the analysis.

Figure 1. .

### Policy analysis (document review)

62 policy documents were analyzed, distributed among 16 different types or categories (Table 1).

**Table 1:** Type or category of document by country (number and percentage)

Category (i)	Chile	Colombia	Ecuador
Resolution		✔	✔
Protocol	✔		✔
Circular		✔	
Guide		✔	✔
Law	✔		
Presidential decree		✔	✔
Ministerial agreement			✔
Guidance recommendation			✔
Technical guideline			✔
Plan of action	✔	✔	
Parliamentary technical assessment	✔		
Technical report	✔		
Technical orientation material		✔	
Ordinary resolution	✔		
Judgment of the Constitutional Court			✔
Total no. of documents = N (%)	**19 (31)**	**25 (40)**	**18 (29)**

*N* = 62

(i) One country can have more than one document per category marked

56% corresponded to documents that addressed the population or the health system in general, and 44% were specific to maternal, perinatal and/or reproductive health care. 85% needed to by complied with obligatorily. Table 2 describes the number and percentage of documents according to their characteristics.

**Table 2:** Number and percentage of documents according to general characteristics by country

Country	Total documents	*N* (%) General (i)	*N* (%) Specific (ii)	*N* (%) Obligatory (iii)
Chile	19	8 (42)	11 (58)	17 (89)
Colombia	25	21 (84)	4 (16)	25 (100)
Ecuador	18	6 (33)	12 (67)	11 (55)
Total	**62**	**35 (56)**	**27 (44)**	**53 (85)**

*N* = 62

(i) for the population at large, (ii) refer specifically to maternal, perinatal and/or reproductive health, (iii) mandatory

Among the general measures, in the three countries lockdowns were established with different modalities depending on the evolution of the pandemic and the need for economic reactivation. There were also special plans or reinforcements of existing lockdowns to alleviate the effects of job loss, under the modality of unconditional cash transfers. Political decisions were communicated through different means. Different communication platforms were used as tools for dissemination and response to the population’s misgivings, such as web pages, social networks, special lines, television, and radio information, among others. Education of the population in self-care measures, such as physical distancing, hand washing, and the use of masks, was extensive, but communication with women and specifically pregnant women was less common. It is also observed that the closure or the limitations of face-to-face care in first level services were not adequately communicated, and that the orientation on the new routes and modalities of care were erratic and changing.

Regarding the policies or specific guidelines for the health sector, the three countries made the decision to close the first level services totally or partially (and second level, in some cases), and concentrated almost all attention on hospital emergency services. In addition, the facilities of the public network were asked to prioritize financial resources, human resources and other measures deemed necessary to deal with the emergency. The centralization of care in emergency services was both for people infected with COVID-19 and for other vital emergencies, such as labor and delivery. The focus of the policies to increase the availability, access, and use of health services in the face of the increase in demand due to the pandemic was the expansion and/or conversion of services, not only in infrastructure (including campaign type hospitals), but also in highly complex equipment, inputs, human resources, and the incorporation of technologies, mainly information and communication technologies (ICT). Telecare was a common innovative element, combined with home visits, follow-up care, and intramural and extramural strategies to promote access and safety for users, even, in some cases, considering the most vulnerable ethnic groups and migrant women. One of the countries partially included educational community visits by health personnel and the taking of laboratory samples at home.

Regarding quality of care, most of the policies or guidelines included general or specific guidelines and/or recommendations with a scientific basis available at the time of their formulation, leaving implicit the possibility of ongoing updates, without clearly specifying the updating and implementation mechanisms. In addition, regarding the training and supervision of health personnel, few documents specify their mechanisms, and only for certain programs. The coordination and continuity of care as strategic elements of primary health care is also an issue addressed in some documents, either through mechanisms such as referral or counter-referral, or the mention of the use of ICT in health establishments to provide advice and monitoring of patients, including pregnant women. Some documents also report information systems mainly for registration and monitoring of vaccination for COVID-19, or to know the evolution of cases and occupation in health services, but clear mechanisms are not considered to ensure follow-up, monitoring and evaluation of the quality and safety in the provision of health services. No specific and systematic records of maternal, perinatal, or reproductive health care (including abortion or post-abortion care) were identified, nor the preservation of respectful care during delivery and the postpartum period.

### Interviews

Some contingencies that arose in the field work resulted in a smaller than planned sample of relatives of deceased women; thus 29 of the 60 planned interviews with relatives were achieved. Eighty semi-structured interviews were carried out that included 21 decision makers, 30 providers and 29 family members (Table 3).

**Table 3:** Planned and actual sample by country

		Chile				Colombia				Ecuador				
		Planned sample		Actual sample		Planned sample		Actual sample		Planned sample		Actual sample		
Interviews with decisionmakers		7		7		7		7		7		7		
Interviews with providers		10		10		10		10		10		10		
Interviews with family members		20		4		20		21		20		4		
Total		**37**		**21**		**37**		**38**		**37**		**21**		

Total N in actual sample = 80

## Results presented according to the three delays

### First delay: recognition of problem and decision to seek care

The interviewees report situations related to the family environment and others in the community environment, with the health system and policies aimed at addressing the pandemic. When the deaths were related to COVID-19, there was a lack of recognition of warning signs associated with the infection. Interviews with relatives of women who died from these causes reported that the decision to seek care was made only in the event of sudden severe symptoms, such as shortness of breath. In cases of maternal deaths not related to COVID-19, women and their families were generally better informed about (obstetric) warning signs, either bleeding or hypertension, that motivated the decision to seek care. Decision makers and providers said that the fear of women and their families to go to health establishments, considering them sources of COVID-19 contagion, could have delayed the search for care, including that which is not considered an emergency, such as routine prenatal consultations. They also identified that the home visits designed in the policies did not have the expected result, either due to a lack of human resources in the health system or due to the refusal of families to be visited for fear of contagion. One of the countries carried out a national campaign on COVID-19 that recommended not going to the care centers, to discourage contagion and overburdening of the centers, which could have influenced the underestimation of signs and symptoms and delayed the decision to consult. In all three countries, decision-makers and providers indicated that domestic work traditionally assigned to women increased considerably during the pandemic due to the permanent presence of children and family members in the home. This situation could have affected the possibility of seeking care and dedicating time to care during pregnancy or postpartum. Furthermore, in one of the countries, the providers indicated that the presence of the partner in the home could have increased the episodes of gender-based violence, which could have affected the decision to go to a health center. The restriction of primary care affected the quality of prenatal care and the possibility of offering useful information for the early detection of symptoms and the eventual need for a consultation, including interventions to counter misinformation regarding COVID-19. During the partial opening, these problems persisted, according to some interviewees.

Countries proposed measures such as home visits and telemedicine to reduce the first delay. In some cases, the creation of local polyclinics was managed, extended prescriptions were delivered, and remote maternity preparation workshops were arranged. However, the interviewees from the three countries indicate that there were gaps in access to technology, both due to infrastructure and digital education, connectivity, lack of preparation and skills of health teams, as well as lack of adequate resources. These interventions also had an impact on the second delay.

### Second delay: seeking care and accessing health services

Relatives of deceased women and members of health teams mentioned that the difficulties generated by the lockdown policies were major obstacles to travel, especially if they did not have their own means of transportation. It was also recognized that many poor families faced an even more precarious economic situation that prevented them from paying for transportation or ability to reside where they would have found medical care. They reported the need to make unforeseen movements wandering from service to service to obtain care and with it, the increase in out-of-pocket expenses. Many establishments stopped providing these services and redirected patients to other units. The decision-makers and providers agree that clear and timely information was not provided to people due to the closure of outpatient services and/or reorganization of the route to follow within the healthcare network. Additionally, some families stated that the deceased woman had consulted several times and that she had not been attended due to the saturation of services.

### Third delay: response of the health services

All the interviewees agree that this was the most significant delay for maternal deaths and other negative outcomes, such as the increase in unintended pregnancies and the increase in fetal deaths.

According to decision-makers and providers, the availability and quality of maternal and reproductive health care in public and private facilities decreased drastically in the first months of the pandemic due to situations that weakened the response capacity of the system. Among them, they mention a greater fragmentation of care to the detriment of comprehensive care and the provision of different studies in a single visit and the concentration of almost all care in the emergency department, without prioritizing pregnant women. Many of the recommended practices for appropriate and respectful maternity care were removed or significantly reduced, thus limiting the quality of care. In the same way, the families reported not only the restrictions for communication with the women and the monitoring of childbirth, but during illness and even death. In some cases, early postpartum discharge was even promoted. These measures also decreased the opportunity to provide information regarding potential complications in pregnancy and the puerperium.

Decision-makers and providers indicate that the general orientation of the health authorities to prioritize care for COVID-19 resulted in many units at different levels of care deciding to close outpatient services, which affected the availability of prenatal and postnatal check-ups. family planning consultations and specialized obstetric consultations, including essential medicines. In one of the countries, contraceptive consultations were particularly affected, and there were no efforts to provide continuity of care through remote care. In another country, the total and/or partial closure of primary health care services and control and monitoring programs was combined with a delay in establishing clear procedures and protocols for maternal, perinatal, and reproductive health care. In a third country, the facilities that normally provided maternity care at the first and second levels stopped doing so and were reorganized following the directive of the health authorities to concentrate the care of deliveries with COVID-19 and serious complications in specifically designated units in some maternity hospitals in each city. This reorganization caused the collapse of second and third level establishments due to overdemand, while the first level was limited to the referral of symptomatic patients to higher levels (when there was availability). According to the providers interviewed, all resources were concentrated on strengthening the second and third levels for COVID 19, and the needs that existed in the first level, including essential medicines, were neglected.

The shortage of health personnel was critical in most countries, either due to redistribution, absenteeism, or vulnerability and fear of contagion. One country even found itself in need of hiring non-graduate personnel.

Decision makers and providers from one of the countries identified some actions that could have reduced the impact in the third delay. Among them, the use of digital information platforms to update knowledge about COVID-19 for health teams; the strengthening of supervision mechanisms for the prevention of infections associated with health care.

### Tracing the formulation of policies to their implementation

In Table 4 we summarize the main gaps between policies and practices and their potential explanations.

**Table 4:** Summary of policy implementation gaps and contributing reasons for the gaps

Level of care (three delays)	Policy goal	Policy Implementation gaps (analyzed through SURE model)	Contributing reasons for gaps
Risk factor recognition and decision to seek care	Emphasize early symptom detection with emphasis on clear, timely communication	Inadequate communication of new guidelines, care routes, and COVID-19 information hindered recognition of warning signs and decisions to seek care; fear of infection delayed care-seeking; the need for women to care for others delayed their own care seeking.	Lack of adequate resources for public health campaigns; fear of contagion.
Accessing healthcare services	Facilitate timely access to traditional site-based care, including prenatal care Establish/expand telehealth	Lockdowns and system restructuring created significant barriers in availability and access, especially for resource-poor individuals, including access to quality reproductive health care. Telehealth implementation proved ineffective; poor access, especially for vulnerable populations, including migrants.	Impact of lockdowns; fragmented public health system hindered coherent response; inadequate resources allocated to implement policies. Inadequate analysis of the system changes needed for effective telehealth implementation.
Healthcare system response	Maintain adequate quality of maternal and reproductive healthcare Promote psychosocial health and respectful maternity care	COVID-19 response prioritized response to care for those infected over maternal care and primary health care leading to a significant decline in the quality of primary care, including maternal and reproductive health services. Many recommended best practices eliminated or significantly reduced.	Resource prioritization towards COVID-19 emergency response and away from routine essential services; inadequate staffing, training and supervision leading to system overload. Resource limitations; staff shortages; altered care priorities.

## Discussion

There is no doubt that the COVID-19 pandemic has had negative effects on the health of pregnant women and numerous studies agree that the MMR has increased in a range between 8.5% and 65.5% [2, 18].

The countries included in this study, like many others, based their policies on implementing strong mobility restrictions to curb the transmission of the virus [19], which led to the total or partial closure of primary care centers and concentrated care in centers of greater complexity. The prioritization of care for people with COVID-19 caused a drastic change in the maternal care model, by redistributing services and human resources (including obstetrics) or by causing the closure or reduction of sexual and reproductive health services [20]. Consistent with other studies, all this affected access, availability, utilization, and quality of care [21] and exposed women and girls to preventable health risks [3, 22], which also violated their rights [23, 24].

Governments made efforts to fill these gaps, and implemented strategies such as telemedicine, home care, and provision of medications with extended coverage (antihypertensives, contraceptives, etc.). However, in line with what has been found in other studies, low digital literacy, connectivity problems and lack of equipment were important barriers [23, 25]. In addition, in the case of telemedicine, the difficulties in outpatient management of pregnant women stand out (even more so with obstetric risks) derived from the impossibility of performing physical examinations and some diagnostic methods [26, 27].

The dissemination of prevention measures and resources available in case of emergencies, mainly through telephone lines, were policies perceived by the interviewees as facilitators to reduce the first and second delays but evaluated as insufficient to generate “alerts” that encourage the women to seek care.

It has been seen that, for the most part, the formulated policies were not accompanied by precise communication strategies [24]. This study shows, in line with other research, that communication problems included the lack of dissemination and diffusion of up-to-date scientific evidence [28], both at the community and health service levels, including that necessary to neutralize false statements about the pandemic [29].

Families of deceased women reported significant barriers to timely, quality maternal healthcare during the pandemic. Suboptimal care resulted from overwhelmed emergency services, communication failures, and resource shortages, particularly for vulnerable populations. Lockdowns further restricted access, and even with access, referral delays were common. Underestimation of the severity of symptoms, fear of hospitalization and mistreatment, and poor communication contributed to the decision of women and their families to delay care. As pointed out by a survey of pregnant women at the beginning of the pandemic, a contributing factor likely was the poor level of knowledge about COVID-19, especially among pregnant women of low socioeconomic status [30]. Inadequate attention to psychosocial and mental health further compromised the quality of care provided. Studies from other countries also reported the negative impact of the pandemic on mental health during pregnancy and the postpartum period 1,2

Additionally, domestic violence increased during the pandemic [3, 31], a phenomenon identified by interviewed providers (but not from the families) as a possible contributor to the increase in maternal deaths, and which has been shown in other studies on pregnant women [32, 33]. At the domestic level, the health context accentuated the high degree of feminization of daily reproduction and care tasks, exacerbating a pre-existing trend in the region [34]. Women generally assume primary or exclusive care, which affects their ability to work [23]. The interviewees reported situations of maternal death in which the woman was caring for others, often as single parents and did not have the resources to get to a health center in the face of a complication.

According to the interviewees, the training and supervision of health personnel was insufficient. Regarding the quality of delivery and newborn care, the study identified that many of the recent gains in respectful care were lost during the pandemic, coinciding with the findings of other studies [35–37]. Regarding supplies, their lack was reported in some cases, as well as the lack of medicines for emergency obstetric care, a lack common to many countries at the beginning of the pandemic [38].

Special situations, not directly related to health institutions, such as home delivery care promoted by a country during an emergency, should also be regulated in these situations, as occurred in some countries that implemented low-risk home delivery policies [39]. Finally, it should be noted that almost no country contemplated guidelines to facilitate access and quality of abortion care.

Despite broadly similar overarching pandemic response strategies (e.g., lockdowns) across the three countries, variations in implementation details (duration, stringency, and financial support), specific strategies (resource allocation, communication methods, resort to telemedicine, and community health worker programs), and contextual factors (socioeconomic disparities, health system structure, digital literacy, and political and cultural contexts) all appeared to influence outcomes. Comparative analysis by country was not possible, as participating countries requested anonymity. However, each country received the complete report, providing valuable information for them to prepare for and respond to future pandemics.

### Limitations

This study had several operational and methodological limitations. In two countries, achieving the target number of family interviews proved difficult due to challenges in obtaining accurate contact information. This was additionally complicated by the ethics committee requirements in one country (i.e., not contacting family members during a months-long mourning periods and mandatory involvement of mental health professionals). Further, the ongoing mobility restrictions during fieldwork affected the team’s ability to contact families. The fact that there were a small number of maternal deaths precluded doing disaggregated analyses by cause or by shorter time periods. Finally, the participating countries’ request for anonymity precluded comparative analysis across countries.

## Implications for practice and policy

Strategies to prioritize in preparing for and responding to similar future pandemics, in relation to protecting maternal and reproductive health, should include effective public awareness campaigns and ensuring continued access to prenatal care; mitigation of access barriers caused by lockdowns and resource limitations; ensuring adequate resource allocation for maternal and reproductive health services and the restoration of best practices for respectful care; and improved strategies for clear and timely communication with both the public and healthcare providers.

Strengthening the response capacity of the health system should not only be considered to deal with health emergencies, but also in “everyday” situations, since many of the gaps are linked to the structural deficits of the health systems and health policies, which the pandemic merely highlighted.

## Supplementary Information

Below is the link to the electronic supplementary material.

Supplementary Material 1

Supplementary Material 2

Supplementary Material 3

Supplementary Material 4

Supplementary Material 5

Supplementary Material 6

Supplementary Material 7

## Data Availability

Data is available upon request; please contact Jim Ricca for more information. The following supplementary materials have been made available: Supplementary file – quantitative analysis. Supplementary file 1a, 1b: Search strategies and Interviewee samples. Supplementary file 2 a-d: Data collection tools. Supplementary file 3: Graphics of excess maternal mortality.

## Abbreviations

UNFPA: United Nations Population Fund
ICT: Information and Communication Technology
PAHO: Pan-American Health Organization

## Footnotes

1 Worrall S, Pike O, Christiansen P, Jackson L, De Pascalis L, Harrold JA, Fallon V, Silverio SA. Psychosocial experiences of pregnant women during the COVID-19 pandemic: a UK-wide study of prevalence rates and risk factors for clinically relevant depression and anxiety. J Psychosom Obstet Gynaecol. 2025 Dec;46 [1]:2,459,619. doi: 10.1080/0167482X.2025.2459619. Epub 2025 Feb 11. PMID: 39,932,229.

2 Maloney CA, Trimm A, Miller-Graff LE. Exploring women’s postpartum experiences during the COVID-19 pandemic: A mixed-method analysis. Midwifery. 2025 Apr;143:104311. doi: 10.1016/j.midw.2025.104311. Epub 2025 Jan 27. PMID: 39,892,225.
