# Supplementary material for: Rapid assessment of the factors contributing to the increase in maternal mortality during the COVID-19 pandemic in the Latin American region
Source: BMC Pregnancy Childbirth. 2026 Jan 3;26:72. doi: 10.1186/s12884-025-08069-y (PMC12828971; doi:10.1186/s12884-025-08069-y)
Supplement: Supplementary file 1 — Supplementary Material 1 [file 12884_2025_8069_MOESM1_ESM.docx]

INTERVIEWEE SAMPLES

**Supplementary Table 1 - Colombia**

|  | **Planned Sample** | | | **Actual Sample** | | |
| --- | --- | --- | --- | --- | --- | --- |
| **Variable** | **Category** | | **Frequency** | **Category** | | **Frequency** |
| **DECISION MAKERS IN SEXUAL AND REPRODUCTIVE HEALTH (N=7)** | | | | | | |
| Department | National | | 3 | National | | 2 |
|  | Bogotá | | 1 | Bogotá | | 2 |
|  | Quindío | | 1 | Cauca Valley | | 1 |
|  | Nariño | | 1 | Valledupar | | 1 |
|  | Vichada | | 1 | Antioquia | | 1 |
| Occupation | Prevention and promotion of MSPS |  | 1 | Prevention and promotion of MSPS |  | 1 |
|  | Contributory EAPB representative | | 1 | Contributory EAPB representative | | 1 |
|  | Subsidized EAPB representative | | 1 | Subsidized EAPB representative | |  |
|  | Hospital directorate |  | 1 | Hospital directorate |  | 2 |
|  | MM and Perinatal Committee Member | | 2 | MM and Perinatal Committee Member | | 2 |
|  |  | |  | Unit leader of maternal and child care | | 1 |
| **PROVIDERS OF MATERNAL, PERINATAL AND REPRODUCTIVE HEALTH SERVICES (N=10)** | | | | | | |
| Department | Boyaca | | 2 | Antioquia | | 4 |
|  | Cauca Valley | | 2 | Cauca Valley | | 2 |

|  | Guajira | 1 | Guajira | | | 1 |
| --- | --- | --- | --- | --- | --- | --- |
|  | Santander | 1 | Bogotá, D.C. | | | 1 |
|  | Chocó | 1 | Chocó | | | 2 |
|  | Norte de Santander | 1 |  | | |  |
|  | Atlantic | 1 |  | | |  |
|  | Bolivar | 1 |  | | |  |
| Occupation | Obstetrician | 4 | Obstetrician | | | 5 |
|  | General Practitioner | 3 | General Practitioner | | | 1 |
|  | Nurse | 3 | Nurse | | | 4 |
| **WOMEN WHO DIED DURING PREGNANCY, CHILDBIRTH OR PUERPERIUM IN THE STUDY PERIOD (N=21)** | | | | | | |
| Age | Less than 20 | At least |  | | |  |
| (Mean= 28.3; |  | 2 | Less than 20 | | | 2 |
| Standard deviation= |  |  | 20 a 29 | | | 9 |
| 6,6) |  |  | 30 a 39 | | | 9 |
|  |  |  | 40 or more | | | 1 |
| Geographical location | Rural | At least 4 | Rural | | | 6 |
|  |  |  | Urban | | | 15 |
| Department |  |  | Antioquia | | | 9 |
|  |  |  | Bogotá, D.C. | | | 2 |
|  |  |  | Boyaca | | | 2 |
|  |  |  | Cauca | | | 1 |
|  |  |  | Cauca Valley | | | 7 |
| Marital Status |  |  | Free Union / Married |  |  | 21 |
|  |  |  | Yes | | | 4 |

| Did you recognize yourself as part of an ethnic group? |  |  | No | 17 |
| --- | --- | --- | --- | --- |
| Country of birth |  |  | Colombia | 19 |
|  |  |  | Venezuela | 2 |
| Educational Level |  |  | Completed primary school | 1 |
|  |  |  | Incomplete primary school | 4 |
|  |  |  | Completed high School | 9 |
|  |  |  | Incomplete high school | 3 |
|  |  |  | Completed Tertiary/university | 4 |
| Stable employment relationship |  |  | No | 18 |
|  |  |  | Yes | 3 |
| I received any subsidy |  |  | No | 15 |
|  |  |  | Yes | 6 |
| Health insurance |  |  | No | 4 |
|  |  |  | Yes | 17 |
| Location of  Death | Institutional |  | Institutional | 20 |
|  | Non-institutional | At least 2 | Non-institutional | 1 |
| Maternal death associated with COVID | Yes | 10 | Yes | 10 |
|  | No | 10 | No | 11 |

Supplementary Table 2: Chile

| Code | Profile |
| --- | --- |
| Decisors | |
| D1 | MT. High-level decision maker. Its relevance to the interview is adjusted in its role for the reorganization of **open care** services. |
| D2 | MT. High-level decision maker. Its relevance to the interview is adjusted in its role for the reorganization of **closed care** services. |
| D3 | MT. Deciding at the local level. In the northern central macro-zone; during the course of the pandemic, COVID-19 strains different from the national behavior could be alerted; in addition, territorially it has urban and rural sectors. |
| D4 | MT Deciding at the local level. The Tarapacá Region had a major migration crisis during 2020-2021, with unknown impact on MM. |
| D5 | MT. Local level decision maker. Geographical representation of maternal mortality. |
| D6 | MT. Decision maker at local level. According to quota criteria, direct decision maker in the obstetrics and gynecology service. MM committee member |
| D7 | Sample T: Local decision-maker. According to quota criteria, direct decider in the service in the obstetrics and gynecology service. |
| Suppliers | |
| P1 | Planned sample. Midwife of a rural CESFAM northern macro zone |
| P2 | Planned sample. Midwife of a high complexity hospital in the North macro zone. |
| P3 | Planned sample. Midwife at the Central Macrozone Hospital. |
| P4 | Planned sample. Surgical unit midwife, deliveries. |
| P5 | Planned sample. Matron of rural sector south macro zone |

| P6 | Planned sample. Physician of a low complexity hospital |
| --- | --- |
| P7 | Planned sample. Medical specialist Hospital Zona Norte |
| P8 | Planned sample. Medical specialist Hospital Metropolitan Region |
| P9 | Planned sample. Medical specialist Hospital Zona Sur |
| P10 | Planned sample. Primary care area general practitioner. |
| Family members | |
| MMF1 | Primiparous, foreign, 26 years old, MM puerperium 19 days, May 2020 |
| MMF2 | Primipara, Chilean, 19 years old, MM late 06 January 2021 |
| GFM3 | Multiparous, Chilean, 28 years old, MM puerperium 15 days, 9 October 2020 |
| GFM4 | Multiparous, Chilean 34 years old, MM puerperium, 3 April 2021 |

Supplementary Table 3: Ecuador

| **Subjects**  **respondents** | **Planned sample** | **Actual sample** | **Profile of**  **interviewers** |
| --- | --- | --- | --- |
| Decisors | 7 officials and former officials of the MSP, in managerial positions at the central, district and/or hospital management level. | 7 officials and former officials of the MSP:   - 3 National Directors - 1 National Maternal-neonatal HIV National Manager - 1 Technician-Dir. Quality Assurance - 1 MSP District Chief - 1 Hospital Manager | 1 health physician and 2 social science professionals with experience in qualitative research on health and gender. |
| Suppliers | 10 providers from MOH facilities: a) from different professions related to obstetric care, b) belonging to facilities at different levels of care, c) in areas related to obstetric care, d) from different levels of care, e) in areas related to obstetric care, and f) in areas related to obstetric care.  and urban areas. | 10 suppliers of MOH facilities:   - 5 midwives/obstetricians - 1 gynecologist - 1 emergency physician - 1 family practice physician - 1 nurse midwife - 1 attending physician with hospital functions | 2 social science professionals with experience in qualitative health and gender research |
| Family members or relatives | 10 cases of maternal death related to COVID-19 and 10 unrelated cases.  1 case ˂ 20 years  3 cases ˃ 20 years  At least 2 rural cases; 2 urban cases  At least 1 non-institutional death | 4 cases of maternal death, 2 related to COVID-19 and 2 not related.  All are institutional deaths of women between 21 and 35 years of age; three cases in urban areas and one in rural areas. | 2 social science professionals with experience in qualitative health and gender research |
