# Supplementary material for: Rapid assessment of the factors contributing to the increase in maternal mortality during the COVID-19 pandemic in the Latin American region
Source: BMC Pregnancy Childbirth. 2026 Jan 3;26:72. doi: 10.1186/s12884-025-08069-y (PMC12828971; doi:10.1186/s12884-025-08069-y)
Supplement: Supplementary file 3 — Supplementary Material 3 [file 12884_2025_8069_MOESM3_ESM.docx]

**Annex 2a: ENGLISH - SEMI-STRUCTURED INTERVIEW GUIDE FOR DECISION-MAKERS**

**PRESENTATION**

This interview aims to talk about the impact of the pandemic and the health policy measures that took place during the past year until July 2021, in particular, in the field of maternal-perinatal health and reproductive health.

It is carried out within the framework of a multicenter study involving 5 Latin American countries (Colombia, Chile, Ecuador, the Dominican Republic and El Salvador), with the support of the United Nations Population Fund and MCGL. This study is aimed at analyzing the factors that may have contributed to the increase in maternal mortality observed in several countries of the region in the last year.

**BLOCK 1: SOCIODEMOGRAPHIC DATA AND PROFILE**

● Age

● Sex

● Profession

● Position

● How long have you been in this position?

**BLOCK 2: HEALTH POLICIES IN THE FACE OF THE PANDEMIC**

Let's start by talking about his view on the general contents of health policy to face the pandemic ...

● What, in your opinion, were the most important health policy measures taken from March 2020 to July 2021?

● What do you think of these measures? Were they relevant and timely? Seen in perspective, do you think any other measures should have been taken? What and for what reasons?

**BLOCK 3: POLICY ON MATERNAL AND PERINATAL HEALTH**

Now we will focus on policy on maternal and perinatal health

● Were there any specific measures in relation to maternal-perinatal health care (especially in relation to prenatal control, delivery care, postnatal care, and care for complications of the mother and newborn)?

- in terms of human resources (incentives, conversion, redeployment, incorporation, supervision, training).

- in terms of financing (budget reallocation and allocation of new resources, demand subsidies, supply subsidies, copayments / bonuses / co-insurance / out-of-pocket payments).

- in terms of supplies and equipment (changes in purchasing and distribution mechanisms).

- in terms of communication (actions towards the population and towards health teams).

● Did you consider strategies to sustain access and utilization? Which?

● Has the maternal and perinatal care model been modified (forms of termination of childbirth, accompaniment, length of hospitalization, residences for mothers, joint hospitalization, etc.)?

● In the area of ​​family planning, what type of model was implemented? What kinds of steps were taken to ensure new and continuing users' access to family planning services?

● What do you think of the measures you identified?

- Were they relevant?

- Were they timely?

● From your perspective, what effects / impacts did these measures have on maternal mortality / perinatal mortality?

● Seen in perspective, do you think it would have been necessary to take other measures? Which? Or design the ones that were taken differently? How?

● Seen in perspective, what have been the most significant challenges for maternal and perinatal health policy?

**BLOCK 4: POLICY ON REPRODUCTIVE HEALTH**

Now let's move on to the questions about reproductive health policies ...

- Were there any specific measures taken in relation to reproductive health care, especially related to the availability and access to contraceptive methods and access to abortion allowed by law? What were these measurements? And what did they imply ...

- in terms of financing (budget reallocation and allocation of new resources).

- in the area of ​​human resources (reconversion, redeployment, incorporation, supervision, training).

- in terms of supplies and equipment (changes in purchasing and distribution mechanisms).

- in terms of communication (actions towards the population and towards health teams).

- Did you consider strategies to sustain access to and utilization of reproductive health services? Which?
- Were specific standards of care or recommendations developed for reproductive health care?
- Were there any specific actions taken in relation to postabortion care? What were those measurements?
- From your perspective, what effects / impacts did these measures (if any) have on unintended pregnancies and abortion complications?
- Seen in perspective, do you think it would have been necessary to take other measures? Which? Or design differently the ones that were taken? How?
- Seen in perspective, what have been the most significant challenges for reproductive health policy?

**BLOCK 5: GENERAL ASSESSMENT AND LESSONS LEARNED FROM THE PANDEMIC**

Looking from the present and in perspective, with everything that has been learned from the successes and mistakes made ...

- What are the factors that, in your opinion, may have contributed to the increase in maternal and perinatal deaths in the first year of the pandemic (2020)?
- What do you think have been the main challenges your country has faced in managing maternal, perinatal and reproductive health care in the context of the pandemic?
- What have been the main lessons learned in the management of maternal, perinatal and reproductive health care in the context of the pandemic?
- What things do you think should have been done differently or should not have been done? For what reasons?

Thank and close the interview

**SPANISH: GUIA DE ENTREVISTA SEMIESTRUCTURADA A DECISORES**

**PRESENTACIÓN**

Esta entrevista tiene como objetivo conversar acerca del impacto de la pandemia y las medidas de política sanitaria que tuvieron lugar durante el año pasado hasta julio de 2021, en particular, sobre el campo de la salud materna-perinatal y la salud reproductiva.

Se realiza en el marco de un estudio multicéntrico que involucra 5 países de América latina (Colombia, Chile, Ecuador, República Dominicana y El Salvador), con el apoyo del Fondo de Población de Naciones Unidas y MCGL. Este estudio está orientado a analizar los factores que pudieron haber contribuido al aumento de la mortalidad materna observada en varios países de la región en el último año.

**BLOQUE 1: DATOS SOCIODEMOGRÁFICOS Y PERFIL**

- Edad
- Sexo
- Profesión
- Cargo
- ¿Cuánto hace que se desempeña en ese cargo?

**BLOQUE 2: POLÍTICAS SANITARIAS FRENTE A LA PANDEMIA**

***Comencemos conversando acerca de su mirada sobre los contenidos generales de la política sanitaria para hacer frente a la pandemia …***

- ¿Cuáles fueron, a su entender, las medidas de política sanitaria más importantes que se tomaron desde marzo de 2020 a julio 2021?
- ¿Qué piensa de esas medidas? ¿Fueron pertinentes y oportunas? Visto en perspectiva, ¿Considera que se deberían haber tomado algunas otras medidas? ¿Cuáles y por qué razones?

**BLOQUE 3: POLÍTICA EN SALUD MATERNA Y PERINATAL**

***Ahora nos enfocaremos en la política en salud materna y perinatal***

- ¿Hubo algunas medidas específicas con relación a la atención en salud materna-perinatal (en especial con relación al control prenatal, a la atención del parto, la atención postnatal, y la atención por complicaciones de la madre y neonato)?
- en materia de recursos humanos (incentivos, reconversión, redistribución, incorporación, supervisión, capacitación).
- en materia de financiamiento (reasignación presupuestaria y asignación de nuevos recursos, subsidios a la demanda, subsidios a la oferta, copagos/bonos/co-seguros/pago de bolsillo).
- en materia de insumos y equipamiento (cambios en los mecanismos de compra y distribución).
- en materia de comunicación (acciones hacia la población y hacia los equipos de salud).
- ¿Consideraron estrategias para sostener el acceso y la utilización? ¿Cuáles?
- ¿Se modificó el modelo de atención materna y perinatal (formas de terminación del parto, acompañamiento, duración de la internación, residencias para madres, internación conjunta, etc.)?
- En al área de la planificación familiar, ¿qué tipo de modelo se implementó? ¿qué tipo de medidas se tomaron para asegurar el acceso de las usuarias nuevas y continuas a los servicios de planificación familiar?
- ¿Qué piensa de las medidas que identificó?
- ¿Fueron pertinentes?
- ¿Fueron oportunas?
- ¿Desde su perspectiva, qué efectos/impactos tuvieron estas medidas sobre la mortalidad materna/mortalidad perinatal
- Visto en perspectiva, ¿Considera que hubiera sido necesario tomar otras medidas? ¿Cuáles? ¿O diseñar de forma diferente las que se tomaron? ¿Cómo?
- Visto en perspectiva, ¿Cuáles han sido los desafíos más significativos para la política de salud materna y perinatal?

**BLOQUE 4: POLÍTICA EN SALUD REPRODUCTIVA**

***Ahora pasemos a las preguntas sobre las políticas en salud reproductiva…***

- ¿Hubo algunas medidas específicas tomadas en relación con la atención en salud reproductiva, especialmente vinculadas a la disponibilidad y el acceso a métodos anticonceptivos y el acceso al aborto permitido por la ley? ¿Cuáles fueron estas medidas? Y ¿Qué implicaron …

- en materia de financiamiento (reasignación presupuestaria y asignación de nuevos recursos).

- en materia de recursos humanos (reconversión, redistribución, incorporación, supervisión, capacitación).

- en materia de insumos y equipamiento (cambios en los mecanismos de compra y distribución).

- en materia de comunicación (acciones hacia la población y hacia los equipos de salud).

- ¿Consideraron estrategias para sostener el acceso y la utilización de servicios de salud reproductiva? ¿Cuáles?
- ¿Se elaboraron normas de atención o recomendaciones específicas para la atención de la salud reproductiva?
- ¿Hubo algunas medidas específicas tomadas en relación con la atención postaborto? ¿Cuáles fueron esas medidas
- ¿Desde su perspectiva, qué efectos/impactos tuvieron estas medidas (si hubieron) sobre los embarazos no planeados y sobre las complicaciones derivadas del aborto?
- Visto en perspectiva, ¿Considera que hubiera sido necesario tomar otras medidas? ¿Cuáles? ¿O diseñar de forma diferente las que se tomaron? ¿Cómo?
- Visto en perspectiva, ¿Cuáles han sido los desafíos más significativos para la política de salud reproductiva?

**BLOQUE 5: EVALUACIÓN GENERAL Y LECCIONES APRENDIDAS DE LA PANDEMIA**

***Mirando desde el presente y en perspectiva, con todo lo que se ha aprendido de los aciertos y errores cometidos …***

- ¿Cuáles son los factores que -a su entender- pueden haber contribuido al aumento de las muertes maternas y perinatales en el primer año de pandemia (2020)?
- ¿Cuáles cree que han sido los principales desafíos que su país ha enfrentado en el manejo de la atención de la salud materna, perinatal y salud reproductiva en el contexto de la pandemia?
- ¿Cuáles han sido las principales lecciones aprendidas en el manejo de la atención de la salud materna, perinatal y salud reproductiva en el contexto de la pandemia?
- ¿Qué cosas cree que se deberían haber hecho diferente o no se deberían haber hecho? ¿Por qué razones?

**Agradecer y cerrar la entrevista**
