# Supplementary material for: Rapid assessment of the factors contributing to the increase in maternal mortality during the COVID-19 pandemic in the Latin American region
Source: BMC Pregnancy Childbirth. 2026 Jan 3;26:72. doi: 10.1186/s12884-025-08069-y (PMC12828971; doi:10.1186/s12884-025-08069-y)
Supplement: Supplementary file 4 — Supplementary Material 4 [file 12884_2025_8069_MOESM4_ESM.docx]

**Annex 2b: SEMI-STRUCTURED PROVIDERS INTERVIEW GUIDE**

**PRESENTATION**

This interview aims to talk about the impact of the pandemic and the health policy measures that took place during the past year until July 2021, in particular, in the field of maternal-perinatal health and reproductive health.

It is carried out within the framework of a multicenter study involving 5 Latin American countries (Chile, Colombia, Ecuador, the Dominican Republic and El Salvador), with the support of the United Nations Population Fund. This study is aimed at analyzing the factors that may have contributed to the increase in maternal and perinatal mortality observed in several countries of the region in the last year.

**BLOCK 1: SOCIODEMOGRAPHIC DATA AND PROFILE**

- Age
- Sex
- Profession
- Position
- How long have you been in this position?
- What functions did you perform in the health service where you usually work during the period March 2020-July 2021?
- Were they different from what he had been doing before the pandemic?
- (if applicable) What were the reasons for the change in your duties?

**BLOCK 2: CHANGES IN HEALTH SERVICES AFTER THE PANDEMIC**

Let's start by talking about the measures that were taken or the changes that occurred in the health service in which you work in the context of the pandemic ...

- What were the most important measures and changes that occurred in the service from March, when it was declared, until July of this year in terms of ...

- maternal-perinatal health care (especially in relation to prenatal care, delivery care and postnatal care, and obstetric and perinatal complications)?

- reproductive health care (especially in relation to the availability and access to contraceptive methods and access to abortion allowed by law and to post-abortion care)?

- human resources (incentives, reconversion, redeployment, incorporation, supervision, training, vaccination for COVID-19 and influenza, personal protection elements)?

- supplies and equipment (availability and adequacy)?

- communication (actions towards the user population and towards the health team)?

● What do you think of these measures?

- Were they relevant?

- Were they timely?

● Were new measures implemented for the management of maternal or perinatal complications in the context of a pandemic? If so, what were they?

● Were specific standards of care, protocols or recommendations developed for the care of pregnancy, childbirth, the puerperium, and newborn care? If so, what were they?

● Were there specific trainings on these topics related to the context of pandemic and social isolation?

- What did they consist of? What aspects did they address?

● Were standards of care or specific recommendations developed for the provision of contraceptive methods and access to termination of pregnancy and / or post-abortion care? If so, what were they?

● Were there specific trainings on the provision of contraceptive methods related to the context of pandemic and social isolation?

- What did they consist of? What aspects did they address?

● How were these measures implemented?

- There were differences between the first and second level of care (health center / hospital).

- In your opinion, were adaptations necessary? If so, in relation to what aspects?

● How were they received by the health teams? How were they sustained throughout the pandemic?

● Seen in perspective, do you think it would have been necessary to take any other measures? Which? Or design differently the ones that were taken? How?

**BLOCK 3: THE IMPACT OF THE PANDEMIC**

Let’s now talk about his perspective on the impact of the pandemic and the measures adopted in the service as a result of the changes that occurred in the regulations ...

● What were the impacts on maternal-perinatal health care during the pandemic? In particular ... (try to discriminate between the impact of the pandemic and the impact of changes in the service)

- in access and quality of prenatal care? (number of prenatal checkups, follow-up, evidence-based inquiries, respectful treatment, women's satisfaction, number of qualified personnel, availability of supplies)

- in access and quality of delivery care? (accompaniment, decision on the type of delivery, use of caesarean section, active management of the delivery, respectful treatment, satisfaction of the women)

- in access and quality of postnatal care? (promotion of breastfeeding, immediate attachment, joint hospitalization, accompaniment, joint discharge, respectful treatment, women's satisfaction)

- in access and quality in the management of maternal and perinatal complications? (adherence to guidelines for obstetric emergencies, adherence to regulations during the pandemic, availability of supplies and human resources, referral and counter referral, satisfaction of women)

● What were the impacts on reproductive health care during the pandemic? In particular ...

- in access to contraceptive methods (availability of a basket, use of digital prescription, availability at the first level of care, change of tasks in the health team, satisfaction of women)

- in the access and quality of abortion care allowed by law (remote provision, change of tasks in the health team, availability at the first level of care, availability of supplies, adherence to evidence-based guidelines, women's satisfaction)

- and in post-abortion care? (availability of manual vacuum aspiration (MVA), availability of supplies and human resources, adherence to evidence-based guidelines, women's satisfaction)

● Do you consider that, in one way or another, the measures taken or the changes introduced in health services may have affected the availability, use or access to maternal-perinatal health services?

- What would be the reasons why availability, use and access were affected?

- How much and in what way was access affected?

● Do you consider that, in some way or another, the measures taken or the changes introduced in health services could have affected the quality of care in maternal, perinatal, abortion, FP services?

- What would be the reasons?

- How much and in what way was the quality of care affected?

● In your opinion, what are the factors that may have contributed to the increase in maternal or perinatal deaths in the first year of the pandemic?

**BLOCK 5: LESSONS LEARNED**

Looking from the present and in perspective, with everything that has been learned from the successes and mistakes made ...

● What do you think have been the main challenges that your country's health services have faced in managing maternal health, perinatal health, and reproductive health in the context of the pandemic?

- What things do you think should have been done differently or should not have been done?

- What would be the reasons?

● What have been the main lessons learned in the management of maternal, perinatal and reproductive health care in the context of the pandemic?

Thank and close the interview.

**SPANISH: GUIA DE ENTREVISTA SEMIESTRUCTURADA A PROVEEDORES**

**PRESENTACIÓN**

Esta entrevista tiene como objetivo conversar acerca del impacto de la pandemia y las medidas de política sanitaria que tuvieron lugar durante el año pasado hasta julio de 2021, en particular, sobre el campo de la salud materna-perinatal y la salud reproductiva.

Se realiza en el marco de un estudio multicéntrico que involucra 5 países de América latina (Chile, Colombia, Ecuador, República Dominicana y El Salvador), con el apoyo del Fondo de Población de Naciones Unidas. Este estudio está orientado a analizar los factores que pudieron haber contribuido al aumento de la mortalidad materna y perinatal observada en varios países de la región en el último año.

**BLOQUE 1: DATOS SOCIODEMOGRÁFICOS Y PERFIL**

- Edad
- Sexo
- Profesión
- Cargo
- ¿Cuánto hace que se desempeña en ese cargo?
- ¿Qué funciones desempeñó en el servicio de salud donde habitualmente trabaja durante el período marzo 2020-julio 2021?
- ¿Fueron diferentes de las que venía desempeñando antes de la pandemia?
- (si corresponde) ¿Cuáles fueron los motivos del cambio de sus funciones?

**BLOQUE 2: CAMBIOS EN LOS SERVICIOS DE SALUD A PARTIR DE LA PANDEMIA**

***Comencemos conversando acerca de las medidas que se tomaron o los cambios que se dieron en el servicio de salud en el que trabaja en el marco de la pandemia …***

- ¿Cuáles fueron las medidas y los cambios más importantes que se dieron en el servicio desde marzo, cuando se declaró, hasta julio de este año en materia de …
- atención en salud materna-perinatal (en especial en relación con la atención prenatal, a la atención del parto y la atención postnatal, y por complicaciones obstétricas y perinatales)?
- atención en salud reproductiva (especialmente en relación con la disponibilidad y el acceso a métodos anticonceptivos y el acceso al aborto permitido por la ley y a la atención postaborto)?
- recursos humanos (incentivos, reconversión, redistribución, incorporación, supervisión, capacitación, vacunación para la COVID-19 e influenza, elementos de protección personal)?
- insumos y equipamiento (disponibilidad y adecuación)?
- comunicación (acciones hacia la población usuaria y hacia el equipo de salud)?
- ¿Qué piensa de esas medidas?
- ¿Fueron pertinentes?
- ¿Fueron oportunas?

● ¿Se implementaron nuevas medidas para el manejo de complicaciones maternas o perinatales en el contexto de pandemia? Si es así, ¿Cuáles fueron?

- ¿Se elaboraron normas de atención, protocolos o recomendaciones específicas para la atención del embarazo, el parto, puerperio y atención del recién nacido? Si es así ¿Cuáles fueron?
- ¿Hubo capacitaciones específicas sobre estos temas vinculadas al contexto de pandemia y aislamiento social?
- ¿En qué consistieron? ¿Qué aspectos abordaron?
- ¿Se elaboraron normas de atención o recomendaciones específicas para la provisión de métodos anticonceptivos y el acceso a la interrupción del embarazo y/o atención posaborto? De ser así, ¿Cuáles fueron?
- ¿Hubo capacitaciones específicas sobre provisión de métodos anticonceptivos vinculados al contexto de pandemia y aislamiento social?
- ¿En qué consistieron? ¿Qué aspectos abordaron?
- ¿Cómo fueron implementadas esas medidas?
- Hubo diferencias entre el primer y el segundo nivel de atención (centro de salud/ hospital).
- En su opinión ¿hubo que hacer adaptaciones? Si fuera así, ¿en relación con qué aspectos?
- ¿Cómo fueron recibidas por los equipos de salud? ¿Cómo se las sostuvo a lo largo de la pandemia?
- ¿Visto en perspectiva, considera que hubiera sido necesario tomar algunas otras medidas? ¿Cuáles? ¿O diseñar de forma diferente las que se tomaron? ¿Cómo?

**BLOQUE 3: EL IMPACTO DE LA PANDEMIA**

***Conversemos ahora sobre su perspectiva acerca del impacto de la pandemia y de las medidas adoptadas en el servicio producto de los cambios que se produjeron en las normativas …***

- ¿Cuáles fueron los impactos en la atención de la salud materna-perinatal durante la pandemia?, en particular … *(procurar discriminar entre el impacto de la pandemia y el impacto de los cambios en el servicio)*
- en el acceso y la calidad de la atención prenatal? (número de controles prenatales, acompañamiento, pesquisas basadas en las evidencias, trato respetuoso, satisfacción de las mujeres, número de personal calificado, disponibilidad de insumos)
- en el acceso y la calidad de la atención del parto? (acompañamiento, decisión sobre el tipo de parto, uso de la cesárea, manejo activo del alumbramiento, trato respetuoso, satisfacción de las mujeres)
- en el acceso y la calidad de la atención postnatal? (promoción de la lactancia, apego inmediato, internación conjunta, acompañamiento, alta conjunta, trato respetuoso, satisfacción de las mujeres)
- en el acceso y calidad en el manejo de complicaciones maternas y perinatales? (adherencia a guías para las emergencias obstétricas, adherencia a normativas durante la pandemia, disponibilidad de insumos y recursos humanos, referencia y contrarreferencia, satisfacción de las mujeres)
- ¿Cuáles fueron los impactos en la atención de la salud reproductiva durante la pandemia?, en particular …
- en el acceso a métodos anticonceptivos (disponibilidad de canasta, uso de receta digital, disponibilidad en el primer nivel de atención, cambio de tareas en el equipo de salud, satisfacción de las mujeres)
- en el acceso y la calidad de la atención del aborto permitido por la ley (provisión a distancia, cambio de tareas en el equipo de salud, disponibilidad en el primer nivel de atención, disponibilidad de insumos, adherencia a guías basadas en las evidencias, satisfacción de las mujeres)
- y en la atención posaborto? (disponibilidad de aspiración manual endouterina (AMEU), disponibilidad de insumos y recursos humanos, adherencia a guías basadas en las evidencias, satisfacción de las mujeres)
- ¿Considera que, de alguna forma u otra, las medidas tomadas o los cambios introducidos en los servicios de salud pudieron haber afectado la disponibilidad, la utilización o el acceso a servicios de salud materna-perinatal?
- ¿Cuáles serían los motivos por los cuales la disponibilidad, la utilización y el acceso fueron afectados?
- ¿Cuánto y de qué forma se afectó el acceso?
- ¿Considera que, de alguna forma u otra, las medidas tomadas o los cambios introducidos en los servicios de salud pudieron haber afectado la calidad de atención de los servicios de salud materna, perinatal, aborto, PF?
- ¿Cuáles serían los motivos?
- ¿Cuánto y de qué forma se afectó la calidad de la atención?
- En su opinión, ¿Cuáles son los factores que pueden haber contribuido al aumento de las muertes maternas o perinatales en el primer año de pandemia?

**BLOQUE 5: LECCIONES APRENDIDAS**

***Mirando desde el presente y en perspectiva, con todo lo que se ha aprendido de los aciertos y errores cometidos …***

- ¿Cuáles cree que han sido los principales desafíos que los servicios de salud de su país han enfrentado en el manejo de la atención de la salud materna, salud perinatal y salud reproductiva en el contexto de la pandemia?
- ¿Qué cosas cree que se deberían haber hecho diferente o no se deberían haber hecho?
- ¿Cuáles serían las razones?
- ¿Cuáles han sido las principales lecciones aprendidas en el manejo de la atención de la salud materna, perinatal y salud reproductiva en el contexto de la pandemia?

**Agradecer y cerrar la entrevista**
