# Supplementary material for: Rapid assessment of the factors contributing to the increase in maternal mortality during the COVID-19 pandemic in the Latin American region
Source: BMC Pregnancy Childbirth. 2026 Jan 3;26:72. doi: 10.1186/s12884-025-08069-y (PMC12828971; doi:10.1186/s12884-025-08069-y)
Supplement: Supplementary file 5 — Supplementary Material 5 [file 12884_2025_8069_MOESM5_ESM.docx]

**Annex 2c: SEMI-STRUCTURED INTERVIEW WITH FAMILY MEMBERS**

Interviewer: complete the information below before conducting the interview.

**CASE FILE**

Country:________________________________________________________________

Town / province / state / region: ______________________________________

● Case number: ______

● Name of interviewee: __________________________________________

● Sex: ____

● Name of the woman: ___________________________________________

● Date of death: __________ (dd / mm / yy)

● Age of the woman: _________

● According to death certificate (check the appropriate option):

- Maternal death
- Maternal death related to COVID-19
- It is not known

● Source of obtaining the case (check the corresponding option):

- Death certificate
- MM committee record
- Hospital registry
- Other (specify)

● Contact information / address: ________________________________________

● Result (check the appropriate option):

- Full interview
- Incomplete interview
- Interview rejected
- It was not possible to find a relative / close friend of the woman because they no longer live there
- Empty house / we can't find the residents

**BLOCK 0: ELIGIBILITY OF THE RESPONDENT**

Interviewer: to start the interview you must have obtained the acceptance of the person to participate after reading the informed consent and obtaining the signature (see options).

The following questions are intended to assess whether the respondent is eligible to be interviewed.

Now that you have agreed to talk to me about the death of _____________ (Interviewer: ALWAYS mention the name of the deceased woman and refer to her throughout the interview calling her by her name)

● When did she die she ___________? __________ (dd / mm / yy)

● Where did _____________ die?

- At home
- On the way to _____________
- In another place, namely: _____________
- In a health institution
- Don't know / don't remember

● How is she related to ____________? What were you of her? _____________

● Were you present when she passed away?

- Yes (the next question is skipped)
- No

● How long before she passed away did you see her? When did she last see you?

- One day before
- One week before
- One month before
- More than a month before
- Don't remember

● Who told you about how she passed away?

- Doctor
- Midwife, Midwife, Nurse
- Family
- Neighbor / friend
- No one, the interviewee saw her
- Other
- Don't know / Don't remember

● How long did it take from when _____________ (deceased's name) passed away until you learned that she was deceased?

- One day later (within 24 hours)
- One week later
- One month later
- More than a month later

● Was anyone with her when she passed away?

- Yes
- No, she was alone
- Don't know

● Who was with her when she passed away?

**FILTER**

The interview ends for those cases in which it is clearly seen that the respondent will not be able to answer the questions in the questionnaire. Examples:

● she last saw her 3 months before she died

● she found out a month after the woman had passed away

● She didn’t know who the woman was with when she passed away

THANK AND LEAVE

**BLOCK 1: SOCIODEMOGRAPHIC DATA**

Next, I am going to ask you some questions about the age of ____________, your marital status, pregnancies, children ...

● How old was _____________ when he passed away?

- ____________
- Don't know / don't answer

● Was she pregnant at the time of death?

- Yes
- No
- Don't know

● Did she die after delivery (between the time of birth or before 42 days after delivery)?

- Yes
- No
- Don't know

● Did she die between 42 days and one year after giving birth?

- Yes
- No
- Don't know

● Did she die during and / or after an abortion / pregnancy loss?

- Yes
- No
- Don't know

Now I want to ask you about the health status of ___________ (name of the deceased).

● Do you know if __________ (name of the deceased) had any illness or health problem? (indicate with an X where applicable)

|  | YES | NO |
| --- | --- | --- |
| Asthma (breathing problems) |  |  |
| Cancer |  |  |
| Obesity |  |  |
| Malnutrition |  |  |
| Diabetes |  |  |
| HIV AIDS |  |  |
| Tuberculosis |  |  |
| Heart Problems |  |  |
| High Blood Pressure |  |  |
| Kidney Problems |  |  |

● Do you know if ____________ (name of deceased) smoked?

- Yes
- No
- Don’t know

● Do you know if she used any drugs / alcohol?

- Yes
- No
- Don’t know

● Do you think her death was related to this disease / these diseases?

- Yes
- No
- Don’t know

● Why do you think this was (or was not) related to this disease?

If the certificate or the records indicate that the maternal death was associated with COVID-19, apply this block:

● Has any family member and / or close friend of ___________ (name of the deceased) been hospitalized in the last month to the date of death due to respiratory problems?

- Yes
- No
- Don’t know

● Do you know if ___________ (name of deceased), in the two weeks prior to death, had contact with a person with a confirmed diagnosis of COVID-19 or who was awaiting results?

- Yes
- No
- Don’t know

● Do you know if ___________ (name of deceased), in the two weeks prior to death, had contact with people in places where COVID-19 cases have been confirmed, such as doctor's offices, hospitals, school, etc.?

- Yes
- No
- Don’t know

● Do you know if ___________ (deceased's name) shared her room with more than one person?

- Yes
- No
- Don’t know

● Do you know if ___________ (name of the deceased) used public transport regularly, in the last 15 days before death?

- Yes
- No
- Don’t know

● Did she have any of the following symptoms during the last 15 days before your death? (indicate with an X where applicable)

|  | YES | NO |
| --- | --- | --- |
| Fever |  |  |
| Difficulty breathing |  |  |
| Sore throat |  |  |
| Fatigue |  |  |
| Headache |  |  |
| Stomache ache, diarrea, vomiting |  |  |

● Do you think the death was related to Covid-19? Why do you think it was related (or not) to this disease? (question for the interviewee to develop on their own terms and from her perspective).

**BLOCK 2: OBSTETRIC HISTORY OF THE DECEASED WOMAN**

Now I am going to ask you some questions about ____________ (name of the deceased): her age, marital status, pregnancies or children (if she had a partner), if she had a partner, some information about the partner.

● At the time of death she was (read the options)

- Together, married
- Separated, divorced
- Single, single, did not have a partner
- Don't know

● How many pregnancies did ___________ (name of deceased) have? _____________

● How many pregnancies did __________ (name of deceased) lose? ____________

● How many normal / vaginal / lower deliveries did ________ (name of deceased) have? _____________

● How many caesarean sections did _____________ (name of deceased) have? _____________

● In any of these pregnancies, did she have any of the following complications? (indicate with an X where applicable)

|  | YES | NO |
| --- | --- | --- |
| Blood loss |  |  |
| High blood pressure |  |  |
| Urinary infection / other infection |  |  |
| Worsening of a previous medical problem |  |  |
| She was hospitalized, but I don’t know why |  |  |

● How many children were born alive, even though they later died? __________

● In what locality or area did ________ (name of the deceased) usually live?

● Was she born in __________ (country of study) or was she born in another country?

- she was born in the country of study.
- she was born in a country other than the study in which Spanish is spoken.
- she was born in a country other than the study country, in which the mother tongue is not Spanish.

● If she was born in another country, how long ago did she live in __________ (country of study)? _________

● What language / language did she usually speak __________ (name of deceased)? ______________

● What was the last year of school or college that she passed?

- Incomplete primary
- Complete primary
- Incomplete secondary
- Complete secondary
- Incomplete tertiary / university
- Complete tertiary / university

● How many people did ______________ (name of deceased) live with? __________

● Did __________ (name of deceased) work outside of her home?

- Yes
- No
- Don’t know

● If the answer is Yes, did she have a steady job with pay?

- Yes
- No
- Don’t know

● Do you know if _____________ (name of deceased) received any plans / programs / assistance from the state / government?

- Yes
- No
- Don’t know

● Did ____________ (name of the deceased) or someone in the close family have their own vehicle?

- Yes
- No
- Don’t know
- Did __________ (name of the deceased) have health coverage / health insurance / medical coverage / social work?
- Yes
- No
- Don’t know

● Did ___________ (name of the deceased) identify herself as belonging to or a descendant of an indigenous people?

- Yes
- No
- Don’t know

● Did the house where the woman lived have potable water?

- Yes
- No
- Don’t know

● Did the house where the woman lived have electricity?

- Yes
- No
- Don’t know

● Did ___________ (name of deceased) or does anyone in your family have a cell phone?

- Yes
- No
- Don’t know

● Do you know if the house where she lived had access to the internet?

- Yes
- No
- Don’t know

**BLOCK 3: FIRST DELAY: IDENTIFICATION OF THE PROBLEM / DECISION TO SEEK HELP**

Now I would like us to talk about how ________ (name of deceased) was and how she felt at first ...

● Do you know if ________ (deceased's name) was pregnant or had recently been pregnant when she died?

- Yes
- No
- Don’t know / Don’t remember (Go to Block 4)

● At what time of pregnancy or childbirth or the puerperium did ________ (name of the deceased) die?

- Before the 5th month of gestation
- Between the 5th and 9th month of gestation
- She died in childbirth
- Died after delivery up to 45 days after it occurred
- Died after 45 days of delivery and before 1 year
- Other
- Don't know / don't remember

● How did you get the news of the pregnancy? How did you react? __________________________________________________________________________________________________________________________________________________________________________

● Was the pregnancy, at any time, a cause of concern for her or for those close to her? Why? (response to develop subjective perspective: emotions, fears, sensations)

__________________________________________________________________________________________________________________________________________________________________________

● Has ____________ (deceased's name) ever used something to prevent pregnancy?

- Yes
- No
- Don’t know / Don’t remember

● Do you know if she was caring for / using any method before she got pregnant?

- Yes
- No
- Don’t know / Don’t remember

● Do you know if she wanted to get pregnant at that time?

- Yes
- No
- Don’t know / Don’t remember

● How did you know that she was pregnant?

- she told him/her
- Saw she was pregnant
- A health professional said so (doctor / midwife)
- she was told by a friend / relative
- other

● Do you know if she did anything to terminate the pregnancy in the last month?

- Yes
- No
- Don’t know / Don’t remember

● If Yes, do you know if she was able to consult with someone (check all that apply)?

- Yes, in the health service
- Yes, in community health services
- Yes, with a lady in the area
- No, but she was able to self-manage / bought pills / found out online and had the abortion
- Did not consult with anyone
- Other:
- Don't know / don't answer

● Do you know if she ever got checked for this pregnancy (prenatal visit)?

- Yes
- No
- Don’t know / Don’t remember

● Do you know in which month of your pregnancy the first check-up was done?

- Yes, in month _____
- No
- Don't know / don't remember

● Do you know if it was checked regularly?

- Yes
- No
- Don’t know / Don’t remember

● Do you know what the doctor / midwife (midwife) / nurse (check all that apply) told you?

- she did not get care
- That she was fine / it was normal
- That I had problems
- To go to another place to attend
- That pregnancy terminations were not made there
- That the pregnancy was too advanced to be able to interrupt
- Other
- Don't know / don't remember

● Do you know how she felt about the treatment she received?

- Better than she expected
- Just as she expected
- Worse than she expected
- Don't know / don't remember

● Do you know if in the week before death (if the death is outside a health institution) or in the week before seeking care prior to death (institutional death) ______________ (name of the deceased) had? (Shade the options that apply)

- Pains / cramps that did not pass with common medications
- Severe headache
- Vaginal bleeding / bleeding
- Infection in the vagina (infection below) / fluid leakage
- dizziness
- Fever
- shortness of breath
- Other
- Don't know / don't answer

● At some point, did she think something was wrong and it wasn't right?

- Yes
- No
- Don’t know / Don’t remember

● Did someone else (family, friends, etc.) think something was wrong and ________ (name of the deceased) was not right?

- Yes
- No
- Don’t know / Don’t remember

● Where was _________ (name of the deceased) when she realized that something was wrong with her and it was not right / when someone else thought something was wrong and was not right?

- At her home / at the home of a relative
- On the way to _____________
- In a health center
- Somewhere else: _______________
- Don't know / don't remember

● Was someone with her when she realized something was wrong and was not right / when someone else thought something was wrong and was wrong?

- Yes, she was with _____________________________________________
- No
- Don't know / don't remember

● How long did it take between her when she realized that something was wrong with her / when someone else thought something was wrong and was wrong and she or someone close to her sought help? ______________________

● Tell me how it was - from what you know – during this time, what happened, the woman or her relative did not want to ask for help, they did not consider it necessary or they tried to solve it at home, who decided that it was necessary to consult, when, if there were doubts, if there were situations that helped or complicated more, if they had made other consultations in the health center or hospital, how they treated her, or things that seem important to highlight from that time- (question for the interviewee develop on your own terms and from your perspective). *

**BLOCK 4: ATEMPT TO ACCESS THE HEALTH SYSTEM**

Now I'm going to ask you about what happened since the woman and / or her family member / companion decided to consult until they were able to get to a place that could treat her

● Do you know if there are hospitals, clinics, health centers where people who live around here are treated?

- Yes
- No
- Don’t know / Don’t remember

● Do you know if ___________ (name of the deceased) had ever resorted to that hospital, clinic, health center for any illness or control?

- Yes
- No
- Don’t know / Don’t remember

● Do you know if _____________ (name of the deceased) went to that hospital, clinic or health center during this pregnancy?

- Yes
- No
- Don’t know / Don’t remember

● Do you attend deliveries and / or complications in that hospital, clinic, or health center?

- Yes
- No
- Don’t know / Don’t remember

● How far is the hospital / clinic from where the woman was? _________

● If she arrived, how long did it take her to get there? _________

● How was ___________ (name of deceased) during that time?

- Good / stable
- worsened
- She was anxious
- did not want to go
- other

● When _________ (name of the deceased) or who decided that she had to be taken somewhere, where did she go? How did she manage to get to the hospital / clinic? Did they have difficulty getting around (inability to leave, lack of transportation, cut routes, at night, bad weather, could not / difficulty to move / unconscious, only one person could move)? Were there any other difficulties (with whom to leave the other children, lack of financial resources, an ambulance was needed and there were none)? (question for the interviewee to develop on their own terms and from her perspective).

● Tell me how it was - from what you know - during, what happened, if there were situations that helped or made it more difficult for her to get to the hospital, clinic or health center, if someone could be with her and accompany her, if there were doubts, What things seem important to highlight from that time- (question for the interviewee to develop in their own terms and according to their perspective).

**BLOCK 5: OBTAINING TREATMENT**

Now I want us to talk about the place where she was treated ...

● Was the place where she were finally treated, was it the first place she came to?

- Yes
- No
- Don’t know / don’t remember

• If it was not the first place, was she referred there or did she go on her own?

- Was referred
- Went there on her own

● Was she seen immediately?

- Yes
- No, had to wait ___________ (waiting time)
- Don't know / don't remember

• Was she hospitalized or under observation?

- Yes
- No
- Don’t know / Don’t remember

● Was she ever sent back to the house?

- Yes, for ______________________________________________________ (reason)
- No
- Don't know / don't remember

• How long was she hospitalized? ___________

• During that time, who cared for her? (doctor, midwife, nurse, assistant, other), did they inform you what was happening? What did they tell you she had? What did they tell you about her health? How do you rate the care she received? Treatment, respect, privacy, response to her problem (question for the interviewee to develop on their own terms and from her perspective).

● Could she have been accompanied by relatives and / or close friends?

- Yes
- No
- Don’t know / Don’t remember

● If she managed to deliver, was she accompanied during the delivery, either normal (vaginal) or cesarean section?

- Yes
- No
- Don’t know / Don’t remember

● During the days that she was hospitalized, how often were relatives and / or close friends informed of how she was _____________ (name of the deceased)?

- Every day
- Every other day
- Every three days
- They did not give us reports

● How were they given information (telephone, guard, WhatsApp, other)? _______________________________________________________________________

If she passed away from COVID

● Do you know if _____________ (name of the deceased) had made any inquiries to the hospital / health center in the 15 days prior to her death?

- Yes
- No
- Don’t know / Don’t remember

● Do you know if they had prescribed mandatory preventive isolation?

- Yes
- No
- Don’t know / Don’t remember

● Do you know if she had received any vaccine for Covid-19?

- Yes, she had the _____________ vaccine (complete / incomplete)
- No
- Don't know / don't remember

● Do you know if you had any tests (swab or blood) for Covid-19 in the last 15 days?

- Yes
- No
- Don’t know / Don’t remember

● If the answer is yes, what was the result?

- Positive
- Negative
- Don't know / don't remember

● How long did it take from the first place you were cared for until she passed away? ___________

● During that time, they informed her or her family member what was happening to her. What did they tell you that she had about her? What did they tell you about her health? How do you rate the care she received? Treatment, respect, information, privacy, response to the problem, support for the family / companion (question for the interviewee to develop on their own terms and from her perspective). *

**PERINATAL INFORMATION (for the cases that apply)**

● Was the baby born alive?

- Yes
- No
- Don’t know / Don’t remember

● How many months pregnant was she when she was born? _____

● How old was the baby when she passed away? _____

● How much did she weigh? ___________

- Don't know

● Do you know if she had problems when she was born?

- Yes, she had ______________________________________________________________
- No
- Don't know / don't remember

• Was she hospitalized?

- Yes, because ____________________________________________________________
- No, the baby stayed with ________________________________________________
- Don't know / don't remember

● How did she do afterwards? ______________________________________________________

● Tell me how it was - from what you know – during that time, what happened, if there were situations that helped or further complicated how the baby was, or helped or complicated the care he needed, things seem important to highlight from that time. (Question for the interviewee to develop on their own terms and from her perspective).

**TO END THE INTERVIEW**

● Is there anything else you would like to tell me?

**Thank and close the interview.**

**SPANISH: ENTREVISTA SEMIESTRUCTURADA A FAMILIARES**

***Entrevistador/a: complete la información que sigue antes de realizar la entrevista.***

FICHA DEL CASO

País:___________________________________________________________________

Localidad/ provincia/ estado/ región: ______________________________________

- No de caso: ______
- Nombre del entrevistado/a:__________________________________________
- Sexo: ____
- Nombre de la mujer: ___________________________________________
- Fecha del fallecimiento: __________(dd/mm/aa)
- Edad de la mujer: _________
- Según certificado de defunción (*marque la opción que corresponda*):
- Muerte materna
- Muerte materna relacionada con COVID-19
- No se sabe
- Fuente de obtención del caso (*marque la opción que corresponda*):
- Certificado de defunción
- Registro del comité de MM
- Registro hospitalario
- Otro (especificar)
- Datos de contacto/ domicilio: ________________________________________
- Resultado (*marque la opción que corresponda*):
- Entrevista completa
- Entrevista incompleta
- Entrevista rechazada
- No fue posible encontrar familiar/ allegado de la mujer porque no viven más allí
- Vivienda vacía/ no encontramos a los moradores

**BLOQUE 0: ELEGIBILIDAD DE EL/LA RESPONDENTE**

***Entrevistador/a: para iniciar la entrevista debe haber obtenido la aceptación de la persona a participar luego de la lectura del consentimiento informado y la obtención de la firma (ver opciones).***

***Las siguientes preguntas tienen por objeto evaluar si la/el respondiente es elegible para ser entrevistada/o.***

Ahora que aceptó hablar conmigo acerca del fallecimiento de _____________ (*Entrevistador/a: mencione SIEMPRE el nombre de la mujer fallecida y refiérase a ella durante toda la entrevista llamándola por su nombre*)

- ¿Cuándo murió ___________? __________ (dd/mm/aa)
- ¿Dónde murió _____________?
  - En su casa
  - En el camino hacia _____________
  - En otro lugar, a saber: _____________
  - En una institución de salud
  - No sabe/ no recuerda
- ¿Cuál es su parentesco con ____________? ¿Qué era usted de ella? _____________

- ¿Estaba usted presente cuando ella falleció?
  - Si (se saltea la siguiente pregunta)
  - No
- ¿Cuánto tiempo antes de que falleciera usted la vio? ¿Cuándo la vio por última vez?
  - Un día antes
  - Una semana antes
  - Un mes antes
  - Más de un mes antes
  - No recuerda
- ¿Quién le habló de cómo falleció?
  - Médica/o
  - Partera, Matrona, Enfermera
  - Familiar
  - Vecina/ amiga
  - Nadie, la entrevistada la vio
  - Otro
  - No sabe/ No recuerda
- ¿Cuánto tiempo pasó desde que _____________ (nombre de la fallecida) falleció hasta que usted supo que ella había fallecido?
  - Un día después (dentro de las 24 horas)
  - Una semana después
  - Un mes después
  - Más de un mes después
- ¿Alguien estaba con ella cuando falleció?
  - Sí
  - No, estaba sola
  - No sabe
- ¿Quién estaba con ella cuando falleció?

| **FILTRO**  **Termina la entrevista para aquellos casos en los que se ve claramente que la/el respondiente no podrá contestar las preguntas del cuestionario. Ejemplos:**   - La vio por última vez 3 meses antes de morir - Se enteró un mes después de que había fallecido la mujer - No sabe con quién estaba la mujer cuando falleció   **DAR LAS GRACIAS Y DESPEDIRSE** |
| --- |

**BLOQUE 1: DATOS SOCIODEMOGRÁFICOS**

A continuación, le voy a hacer algunas preguntas sobre la edad de ____________, su estado civil, los embarazos, los hijos…

- ¿Qué edad tenía _____________ cuando falleció?
  - ____________
  - No sabe/ no responde
- ¿Estaba embarazada al momento de la muerte?
  - Sí
  - No
  - No sabe
- ¿Falleció después de un parto (entre el momento del nacimiento o antes de 42 días después del parto)?
  - Sí
  - No
  - No sabe
- ¿Falleció entre los 42 días y un año posterior a un parto?
  - Sí
  - No
  - No sabe
- ¿Falleció durante y/o después de un aborto/ pérdida del embarazo?
  - Sí
  - No
  - No sabe

Ahora quiero preguntarle por el estado de salud de ___________ (nombre de la fallecida).

- ¿Conoce si __________ (nombre de la fallecida) tenía alguna enfermedad o problema de salud? (indique con una **X** donde corresponda)

|  | Si | No |
| --- | --- | --- |
| Asma (problemas respiratorios) |  |  |
| Cáncer |  |  |
| Obesidad |  |  |
| Desnutrición |  |  |
| Diabetes |  |  |
| VIH sida |  |  |
| Tuberculosis |  |  |
| Problemas de corazón |  |  |
| Presión alta |  |  |
| Problemas en los riñones |  |  |

- ¿Sabe si ____________ (nombre de la fallecida) fumaba?
  - Si
  - No
  - No sabe
- ¿Sabe si consumía alguna droga/alcohol?
  - Si
  - No
  - No sabe
- ¿Cree que su muerte estuvo relacionada con esta enfermedad/ estas enfermedades?
  - Si
  - No
  - No sabe
- ¿Por qué cree que estuvo relacionada (o no) con esta enfermedad?

**Si en el certificado o en los registros se indica que la muerte materna estuvo asociada a COVID-19, aplicar este bloque:**

- ¿Algún familiar y/o amigo cercano de ___________ (nombre de la fallecida) estuvo hospitalizado en el último mes a la fecha de muerte por problemas respiratorios?
  - Si
  - No
  - No sabe
- ¿Sabe si ___________ (nombre de la fallecida), en las dos semanas previas a la muerte, tuvo contacto con una persona con diagnóstico confirmado de COVID-19 o que estuviese en espera de resultados?
  - Si
  - No
  - No sabe
- ¿Sabe si ___________ (nombre de la fallecida), en las dos semanas previas a la muerte, tuvo contacto con personas en sitios donde se hayan confirmado casos por COVID-19, tales como consultorios, hospitales, escuela, etc.?
  - Si
  - No
  - No sabe
- ¿Sabe si ___________ (nombre de la fallecida) compartía su habitación con más de una persona?
  - Si
  - No
  - No sabe
- ¿Sabe si ___________ (nombre de la fallecida) utilizaba transporte público habitualmente, en los 15 días últimos previos a la muerte?
  - Si
  - No
  - No sabe

- ¿Tuvo alguno de los siguientes síntomas durante los últimos 15 días antes de su muerte? (indique con una **X** donde corresponda)

|  | Si | No |
| --- | --- | --- |
| Fiebre |  |  |
| Dificultad respiratoria |  |  |
| Dolor de garganta |  |  |
| Fatiga |  |  |
| Dolor de cabeza |  |  |
| Malestar estomacal, diarrea, vómitos |  |  |

- ¿Cree que la muerte estuvo relacionada con el Covid-19? ¿Por qué cree que estuvo relacionada (o no) con esta enfermedad? *(pregunta para que el/la entrevistado/a desarrolle en sus propios términos y según su perspectiva).*

**BLOQUE 2: ANTECEDENTES OBSTÉTRICOS DE LA MUJER FALLECIDA**

Ahora voy a hacerle algunas preguntas sobre ____________ (nombre de la fallecida): su edad, estado civil, los embarazos o hijos (si tenía), si tenía pareja, algunos datos de la pareja.

- En el momento de fallecer estaba (*leer las opciones*)
  - Unida, casada
  - Separada, divorciada
  - Soltera, sola, no tenía pareja
  - No sabe
- ¿Cuántos embarazos tuvo ___________ (nombre de la fallecida)? _____________
- ¿Cuántos embarazos perdió __________ (nombre de la fallecida)? ____________
- ¿Cuántos partos normales/ vaginales/ por abajo tuvo ________ (nombre de la fallecida)? _____________
- ¿Cuántas cesáreas tuvo _____________ (nombre de la fallecida)? _____________
- ¿En algunos de esos embarazos tuvo alguna de las siguientes complicaciones? (indique con una **X** donde corresponda)

|  | Si | No |
| --- | --- | --- |
| Pérdida de sangre |  |  |
| Presión alta |  |  |
| Infección urinaria/ otra infección |  |  |
| Se le agravó una enfermedad que tenía |  |  |
| La internaron y no sé por qué |  |  |

- ¿Cuántos hijos nacieron vivos, aunque después hayan fallecido? __________
- ¿En qué localidad o zona vivía ________ (nombre de la fallecida) habitualmente?
- ¿Ella nació en __________ (país del estudio) o había nacido en otro país?
- Nació en el país del estudio.
- Nació en un país distinto al del estudio en el que se habla español.
- Nació en un país distinto al del estudio, en el que la lengua materna no es el español.
- Si nació en otro país, ¿hace cuánto tiempo vivía en __________ (país del estudio)? _________
- ¿En qué idioma/ lengua hablaba habitualmente __________ (nombre de la fallecida)? ______________
- ¿Cuál fue el último año de escuela o universidad que aprobó?
- Primario incompleto
- Primario completo
- Secundario incompleto
- Secundario completo
- Terciario/universitario incompleto
- Terciario/universitario completo
- ¿Con cuántas personas vivía ______________ (nombre de la fallecida)? __________
- ¿__________ (nombre de la fallecida) trabajaba fuera de su casa?
  - Sí
  - No
  - No sabe
- Si la respuesta es Sí, ¿tenía un trabajo fijo con salario?
  - Sí
  - No
  - No sabe
- ¿Sabe si _____________ (nombre de la fallecida) recibía algún plan/ programa/ ayuda del estado/gobierno?
  - Sí
  - No
  - No sabe
- ¿____________ (nombre de la fallecida) o alguien de la familia cercana tenía vehículo propio?
  - Sí
  - No
  - No sabe

¿__________ (nombre de la fallecida) tenía cobertura de salud/ seguro de salud/ cobertura médica/ obra social?

- - Sí
  - No
  - No sabe
- ¿___________ (nombre de la fallecida) se identificaba como perteneciente o descendiente de algún pueblo originario?
  - Sí
  - No
  - No sabe
- ¿La casa donde vivía la mujer tenía agua potable?
  - Sí
  - No
  - No sabe
- ¿La casa donde vivía la mujer tenía luz?
  - Sí
  - No
  - No sabe
- ¿Tenía ___________ (nombre de la fallecida) o alguien de su familia un teléfono celular?
  - Sí
  - No
  - No sabe
- ¿Sabe si en la casa donde ella vivía había acceso a internet?
  - Sí
  - No
  - No sabe

**BLOQUE 3: PRIMERA DEMORA: IDENTIFICACIÓN DEL PROBLEMA/DECISIÓN DE BUSCAR AYUDA**

Ahora quisiera que habláramos de cómo estaba ________(nombre de la fallecida) y cómo se sintió al principio…

- ¿Sabe usted si cuando falleció ________ (nombre de la fallecida) estaba embarazada o recientemente había estado embarazada?
  - Si
  - No (pasa a bloque 4)
  - No sabe/ no recuerda (pasa a bloque 4)
- ¿En qué momento del embarazo o del parto o puerperio falleció ________ (nombre de la fallecida)?
  - Antes del 5to mes de gestación
  - Entre el 5to y 9no mes de gestación
  - Falleció en el parto
  - Falleció después del parto hasta 45 días después de ocurrido
  - Falleció después de los 45 días del parto y antes del año
  - Otra
  - No sabe/ no recuerda
- ¿Cómo tomó la noticia del embarazo? ¿Cómo reaccionó? _____________________________________________________________________________________________________________________________________________________________________________________________________________________
- ¿El embarazo fue, en algún momento, motivo de preocupación para ella o para sus allegados? ¿Por qué? (respuesta para desarrollar la perspectiva subjetiva: emociones, miedos, sensaciones)

_____________________________________________________________________________________________________________________________________________________________________________________________________________________

- ¿Alguna vez ____________ (nombre de la fallecida) usó algo para no embarazarse?
  - Sí
  - No
  - No sabe/ no recuerda
- ¿Sabe si ella se estaba cuidando/usando algún método antes de embarazarse?
  - Sí
  - No
  - No sabe/ no recuerda
- ¿Sabe si ella quería quedar embarazada en ese momento?
  - Si
  - No
  - No sabe/ no recuerda
- ¿Cómo supo usted que ella estaba embarazada?
  - Ella le contó
  - La vio embarazada
  - Le contó un profesional de la salud (médico/ partera)
  - Le contó una amiga/ familiar
  - Otro
- ¿Sabe si en el último mes hizo algo para interrumpir el embarazo?
- Sí
- No
- No sabe/ No recuerda
- Si responde Sí, ¿sabe si pudo consultar con alguien (marque todas las que corresponda)?
  - Sí, en el servicio de salud
  - Sí, en servicios de salud comunitaria
  - Sí, con una señora de la zona
  - No, pero pudo autogestionar/ compró pastillas/ supo por internet y hacerse el aborto
  - No consultó con nadie
  - Otra:
  - No sabe/ no responde
- ¿Sabe si alguna vez fue a que la revisaran por este embarazo (visita prenatal)?
  - Sí
  - No
  - No sabe/ no recuerda
- ¿Sabe en qué mes del embarazo se hizo el primer control?
  - Sí, en el mes _____
  - No
  - No sabe/ no recuerda
- ¿Sabe si se controlaba regularmente?
  - Sí
  - No
  - No sabe/ no recuerda
- ¿Sabe qué le dijo el médico/ la partera (matrona)/ la enfermera (marque todas las que corresponda)?
  - No logró que la atendieran
  - Que estaba todo bien/ era normal
  - Que tenía problemas
  - Que fuera a atenderse a otro lugar
  - Que allí no se hacían interrupciones de embarazo
  - Que el embarazo estaba muy avanzado para poder interrumpir
  - Otro
  - No sabe/ no recuerda
- ¿Sabe cómo se sentía ella con el trato recibido?

o Mejor de lo que esperaba

o Igual que como esperaba

o Peor de lo que esperaba

- - No sabe/ no recuerda
- ¿Sabe si en la semana antes de fallecer (si la muerte es fuera de una institución de salud) o en la semana antes de buscar atención previa al fallecimiento (muerte institucional) ______________ (nombre de la fallecida) tuvo? (Sombrear las opciones que correspondan)
  - Dolores/ cólicos que no pasaban con medicamentos comunes
  - Dolor de cabeza intenso
  - Hemorragia/ sangrado vaginal
  - Infección en la vagina (infección abajo) / pérdida de líquido
  - Mareos
  - Fiebre
  - Falta de aire
  - Otro
  - No sabe/ no responde
- En algún momento, ¿ella pensó que algo pasaba y no estaba bien?
  - Si
  - No
  - No sabe/ no recuerda
- ¿Otra persona (familiares, amigos/amigas, etc.) pensó que algo pasaba y ________ (nombre de la fallecida) no estaba bien?
  - Si
  - No
  - No sabe/ no recuerda
- ¿Dónde se encontraba _________ (nombre de la fallecida) cuando se dio cuenta que algo le pasaba y no estaba bien/ cuando otra persona pensó que algo pasaba y no estaba bien?
- En su casa/ en casa de un familiar
- En camino hacia _____________
- En un centro de salud
- En otro lugar, _______________
- No sabe/ no recuerda
- ¿Estaba alguien con ella cuando se dio cuenta que algo pasaba y no estaba bien/ cuando otra persona pensó que algo pasaba y no estaba bien?
- Sí, estaba con _____________________________________________
- No
- No sabe/ no recuerda
- ¿Cuánto tiempo pasó entre que ella cuando se dio cuenta que algo le pasaba y no estaba bien/ cuando otra persona pensó que algo pasaba y no estaba bien y ella o alguien cercano buscó ayuda? ______________________
- Cuénteme cómo fue -de lo que usted sabe- todo este tiempo, qué pasó, la mujer o su familiar no quiso pedir ayuda, no lo consideraron necesario o trataron de resolverlo en casa, quién decidió que había que consultar, cuándo, si había dudas, si hubo situaciones que ayudaron o complicaron más, si habían realizado otros consultas en el centro de salud u hospital, cómo la trataron, o cosas que le parecen importantes de destacar de ese tiempo- *(pregunta para que el/la entrevistado/a desarrolle en sus propios términos y según su perspectiva).* *

**BLOQUE 4: BÚSQUEDA DE ACCEDER AL SISTEMA DE SALUD**

Ahora voy a preguntarle sobre lo que pasó desde que la mujer y/o su familiar/acompañante decidieron consultar hasta que pudieron llegar a un lugar que pudieran atenderla

- ¿Sabe si hay hospitales, clínicas, centros de salud donde se atienda la gente que vive por aquí?
  - Sí
  - No
  - No sabe/ no recuerda
- ¿Sabe si ___________ (nombre de la fallecida) había recurrido alguna vez a ese hospital, clínica, centro de salud por alguna enfermedad o control?
  - Sí
  - No
  - No sabe/ no recuerda
- ¿Sabe si _____________ (nombre de la fallecida) fue a ese hospital, clínica o centro de salud durante este embarazo?
  - Sí
  - No
  - No sabe/ no recuerda
- ¿En ese hospital, clínica, centro de salud atienden partos y/o complicaciones ?
  - Sí
  - No
  - No sabe/ no recuerda
- ¿A qué distancia está el hospital/clínica de donde estaba la mujer? _________
- En caso de haber llegado, ¿Cuánto tiempo le llevó movilizarse hasta allí? _________
- ¿Cómo estuvo ___________ (nombre de la fallecida) durante ese tiempo?
  - Bien/estable
  - Empeoró
  - Estaba ansiosa
  - No quería ir
  - Otro
- Cuando _________ (nombre de la fallecida) o quien decidió que había que llevarla a algún lugar, ¿donde acudió? ¿Cómo hicieron para trasladarse hasta el hospital/clínica? ¿Tuvieron dificultades para trasladarse (imposibilidad de salir, falta de transporte, rutas cortadas, de noche, mal clima, no podía/dificultad para movilizarse /inconsciente sólo podía movilizarse una persona)? ¿Hubo dificultades de otro tipo (con quién dejar los otros hijos, falta de recursos económicos, se necesitaba ambulancia y no había)? *(pregunta para que el/la entrevistado/a desarrolle en sus propios términos y según su perspectiva).*
- Cuénteme cómo fue -de lo que usted sabe- todo este tiempo, qué pasó, si hubo situaciones que ayudaron o complicaron más que llegara al hospital, clínica o centro de salud, si alguien pudo estar con ella y acompañarla, si había dudas, que cosas le parecen importantes de destacar de ese tiempo- *(pregunta para que el/la entrevistado/a desarrolle en sus propios términos y según su perspectiva).*

**Sección 5: OBTENCIÓN DE TRATAMIENTO**

Ahora quiero que hablemos del lugar donde la atendieron…

- El lugar donde finalmente la atendieron, ¿fue el primer lugar al que llegaron?
  - Sí
  - No
  - No sabe/ no recuerda
- ¿Si no fue el primer lugar, ¿fue derivada allí o llegaron por sus propios medios?
- Fue derivada
- Fue por sus propios medios
- ¿Fue atendida inmediatamente?
  - Sí
  - No, tuvo que esperar ___________ (tiempo de espera)
  - No sabe/ no recuerda
- ¿Quedó hospitalizada o en observación?
  - Si
  - No
  - No sabe/ no recuerda
- ¿En algún momento la enviaron de vuelta a la casa?
  - Sí, por ______________________________________________________ (motivo)
  - No
  - No sabe/ no recuerda
- ¿Cuánto tiempo estuvo internada? ___________
- Durante ese tiempo, ¿quién la atendió? (médico, matrona, enfermera, auxiliar, otro), le informaron lo que pasaba? ¿Qué le dijeron que tenía? ¿Qué le dijeron sobre su estado de salud? ¿Cómo valora la atención que ella recibió? El trato, respeto, privacidad, respuesta a su problema *(pregunta para que el/la entrevistado/a desarrolle en sus propios términos y según su perspectiva).*
- ¿Pudo estar acompañada por familiares y/o allegados?
  - Sí
  - No
  - No sabe/ no recuerda
- Si alcanzó a tener el parto ¿estuvo acompañada durante el parto, ya sea normal (vaginal) o cesárea?
  - Si
  - No
  - No sabe/ no recuerda
- Durante los días que estuvo internada, ¿cada cuánto tiempo les informaban a los familiares y/o allegados de cómo estaba _____________ (nombre de la fallecida)?
  - Todos los días
  - Día por medio
  - Cada tres días
  - No nos dieron informes
- ¿De qué forma les daban información (teléfono, en la guardia, WhatsApp, otra)? _______________________________________________________________________

Si falleció por COVID

- ¿Sabe si _____________ (nombre de la fallecida) había hecho alguna consulta al hospital/centro de salud en los 15 días previos a que falleciera?
  - Si
  - No
  - No sabe/ no recuerda
- ¿Sabe si le habían indicado aislamiento preventivo obligatorio?
  - Si
  - No
  - No sabe/ no recuerda
- ¿Sabe si le habían recibido alguna vacuna para Covid-19?
  - Si, tenia el esquema _____________ (completo/incompleto)
  - No
  - No sabe/ no recuerda
- ¿Sabe si le habían hecho algún examen (hisopado o sangre) para Covid-19 en los últimos 15 días?
  - Si
  - No
  - No sabe/ no recuerda
- Si la respuesta es sí, ¿Cuál fue el resultado?
  - Positivo
  - Negativo
  - No sabe/ no recuerda
- ¿Cuánto tiempo pasó desde el primer lugar donde la atendieron hasta que falleció? ___________
- Durante ese tiempo, le informaron a ella o su familiar que le pasaba ¿Qué le dijeron que tenía? ¿Qué le dijeron sobre su estado de salud? ¿Cómo valora la atención que ella recibió? Trato, respeto, información, privacidad, respuesta al problema, apoyo a la familia/acompañante *(pregunta para que el/la entrevistado/a desarrolle en sus propios términos y según su perspectiva).* *

**INFORMACIÓN PERINATAL (para los casos que aplique)**

- ¿El bebé nació vivo?
  - Si
  - No
  - No sabe/ no recuerda
- ¿Cuántos meses de embarazo tuvo cuando nació? _____
- ¿Qué edad tenía el bebé cuando falleció? _____
- ¿Cuánto pesó? ___________
  - No sabe
- ¿Sabe si tuvo problemas cuando nació?
  - Sí, tuvo ______________________________________________________________
  - No
  - No sabe/ no recuerda
- ¿Quedó internado?
  - Sí, porque ____________________________________________________________
  - No, el bebé se quedó con ________________________________________________
  - No sabe/ no recuerda
- ¿Cómo siguió luego? ________________________________________________________________
- Cuénteme cómo fue -de lo que usted sabe- todo ese tiempo, qué pasó, si hubo situaciones que ayudaron o complicaron más cómo estaba el bebé, o ayudaron o complicaron la atención que necesitaba, cosas le parecen importantes de destacar de ese tiempo. *(Pregunta para que el/la entrevistado/a desarrolle en sus propios términos y según su perspectiva).*

**PARA TERMINAR LA ENTREVISTA**

- ¿Hay algo más que quisiera contarme?

**Agradecer y cerrar la entrevista**
