# Supplementary material for: Rapid assessment of the factors contributing to the increase in maternal mortality during the COVID-19 pandemic in the Latin American region
Source: BMC Pregnancy Childbirth. 2026 Jan 3;26:72. doi: 10.1186/s12884-025-08069-y (PMC12828971; doi:10.1186/s12884-025-08069-y)
Supplement: Supplementary file 6 — Supplementary Material 6 [file 12884_2025_8069_MOESM6_ESM.docx]

## **Supplementary file 1ª. Document search strategies**

The national research teams conducted the document search based on four main strategies, namely:

1. Key informants: the national teams consulted specialists in the field, both within and outside the national ministries of health; in parallel, the coordinating team carried out a process of review of sources and supervision, which resulted in the suggestion, deliberation and eventual inclusion of documents that had not been initially contemplated.
2. Targeted search in official websites: supervised and/or carried out by the focal points of the national teams, based on the general inclusion criteria established in the specific protocol.
3. Keyword-driven search in documents in pdf format and/or "open" documents on web pages. To define whether the document met the inclusion criteria, it was proposed to search the document, when possible, using the following words keywords:

*Pregnancy (includes pregnant, pregnancy), childbirth, postpartum, neonatal (includes neonate/s, neonatology), prenatal, maternity (includes maternal, maternity), perinatal (includes perinatal), gestation (includes gestation, gestation, gestational), born, birth, lactation, sexual health, reproductive health, voluntary interruption of pregnancy, abortion (includes abortion, abortifacient), family planning, contraception (includes contraception, contraceptive/s).*

*Translated with www.DeepL.com/Translator (free version)*The procedure/statement was that the key word or phrase (in italics) should be placed in the search field of the document. The same instruction was used for the analysis of open documents on Web sites. Given the volume of texts to be analyzed, this procedure was adopted to facilitate the location of the topics and/or population under study within the documents. The following graphs illustrate this type of search according to document format.

Supplementary Figure 1. Truncated terms in a "PDF" file


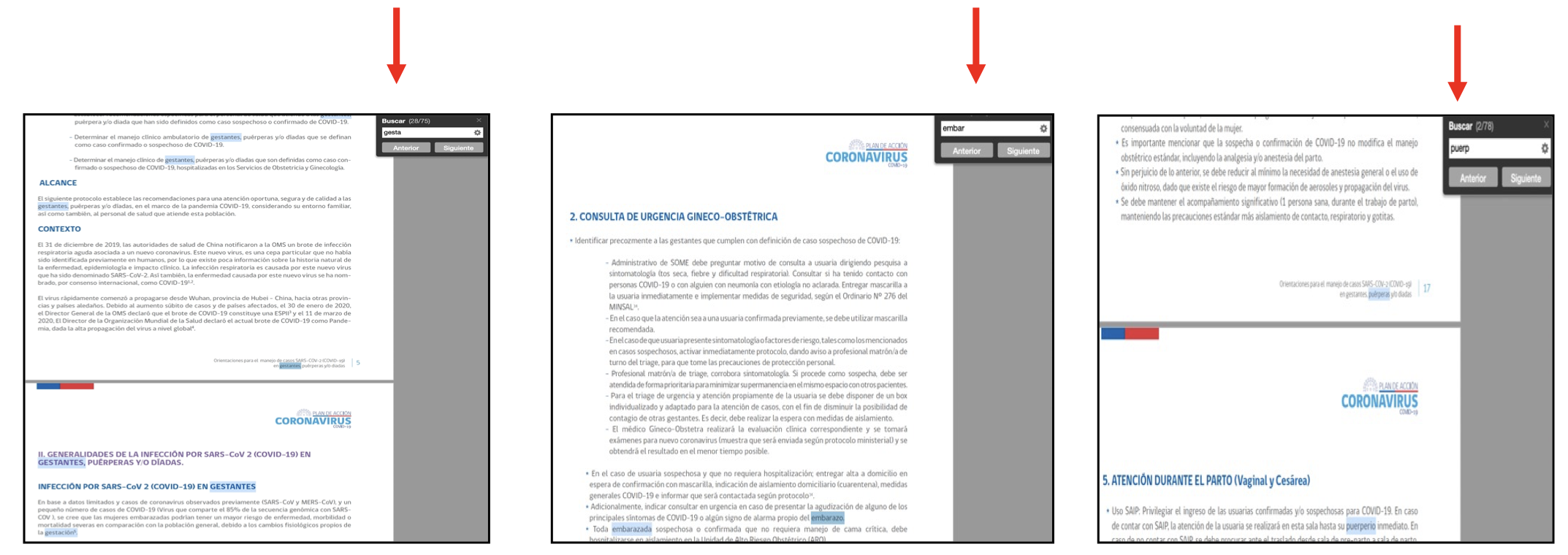


Supplementary Figure 2. Searching Web sites


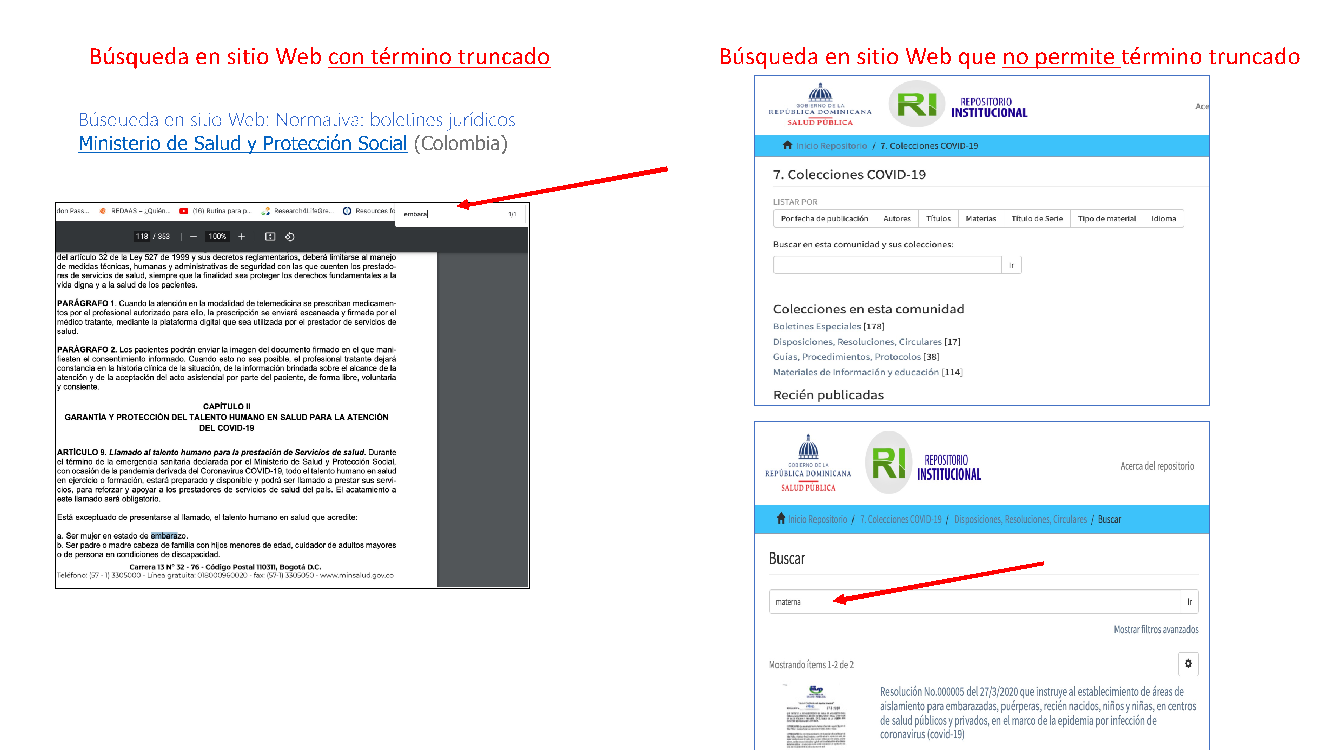


Finally, in those documents where it was not possible to search with key terms, the indication was the reading the document in its entirety and extracting the relevant information.

## **Collection, validation and analysis strategy**

Each country designed the search strategies supervised or carried out by the focal points (i.e. the principal investigator of each team). (i.e., the principal investigator of each team).. The selection of documents was carried out by two members of the team independently, and any doubts regarding their inclusion were resolved by the focal point or the coordination team (CEDES). The uploading was carried out individually and the focal point was in charge of doing the uploading and evaluating it. Any disagreement was resolved locally until a consensus was reached, and, when consensus was not reached, it was resolved with the coordination team (CEDES).
