# Supplementary material for: Rapid assessment of the factors contributing to the increase in maternal mortality during the COVID-19 pandemic in the Latin American region
Source: BMC Pregnancy Childbirth. 2026 Jan 3;26:72. doi: 10.1186/s12884-025-08069-y (PMC12828971; doi:10.1186/s12884-025-08069-y)
Supplement: Supplementary file 7 — Supplementary Material 7 [file 12884_2025_8069_MOESM7_ESM.docx]

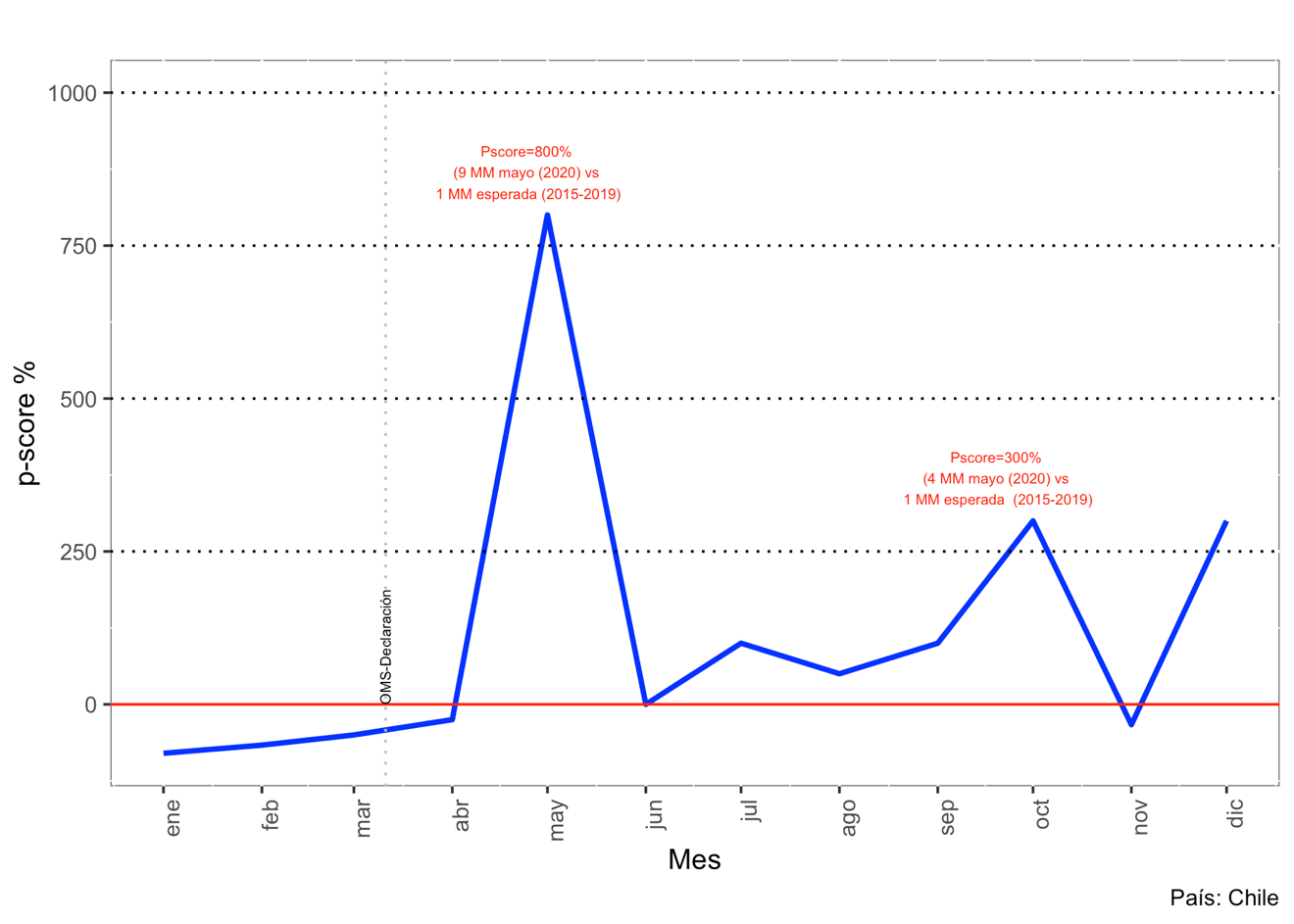


**Supplementary Figure 3: Excess death (p-score). Country: Chile**

**Supplementary Figure 4: Excess death (p-score). Country: Colombia**


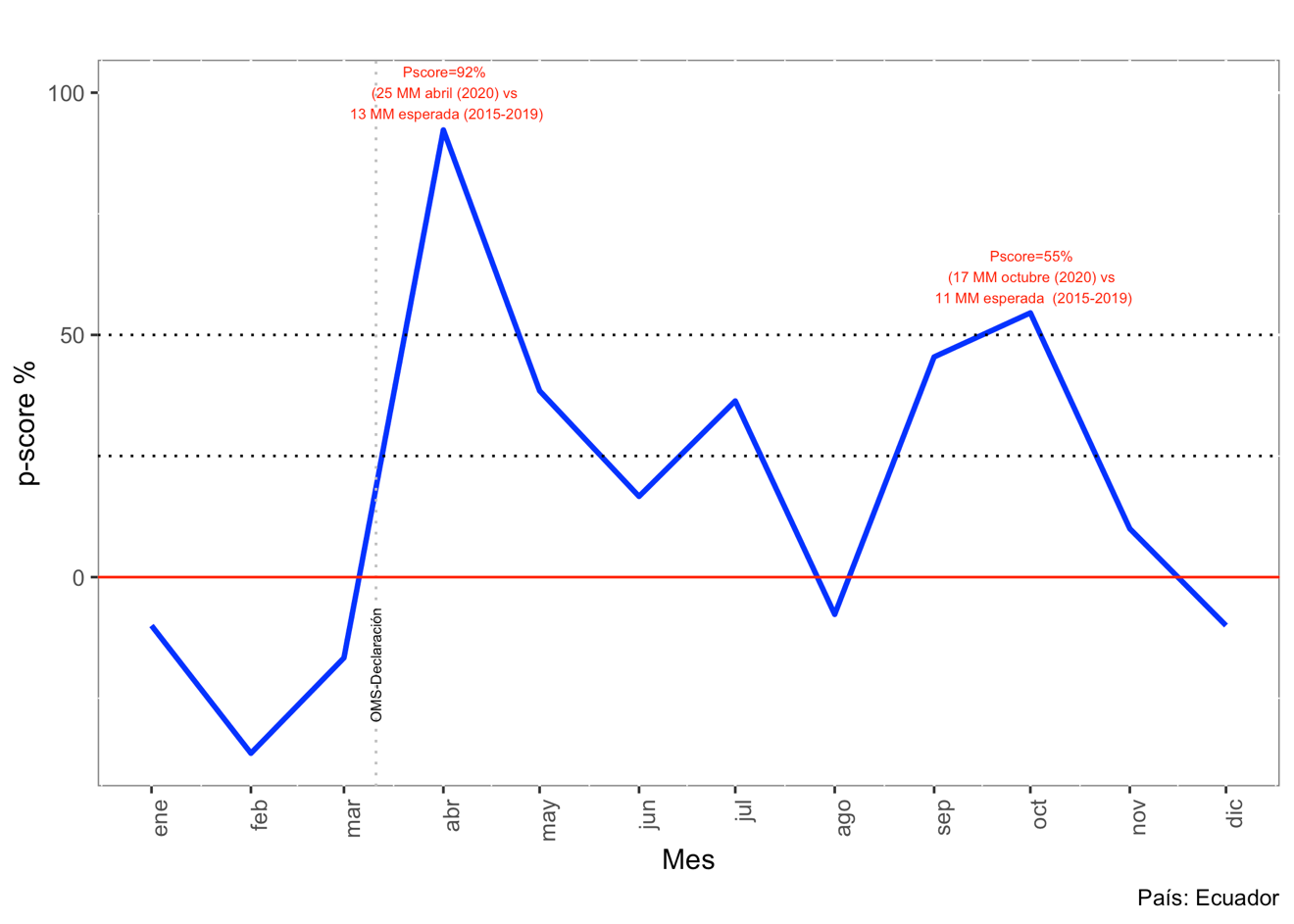


**Supplementary Figure 5: Excess death (p-score). Country: Ecuador**


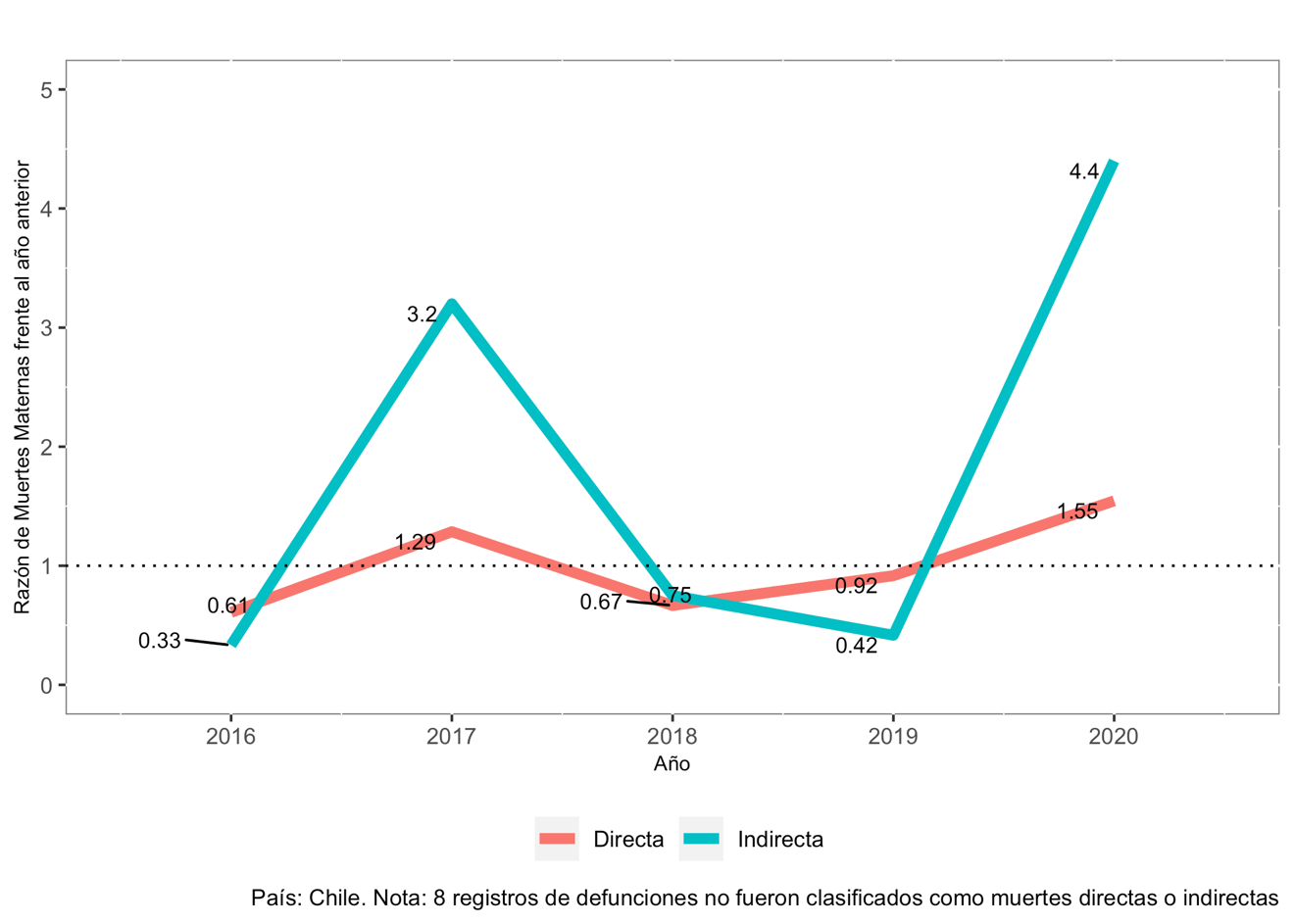


**Supplementary Figure 6: Maternal mortality by classification (direct/indirect). Ratio vs. previous year. 2015-2021. Country: Chile**

**NOTE: 8 registered deaths were not classified as direct or indirect**


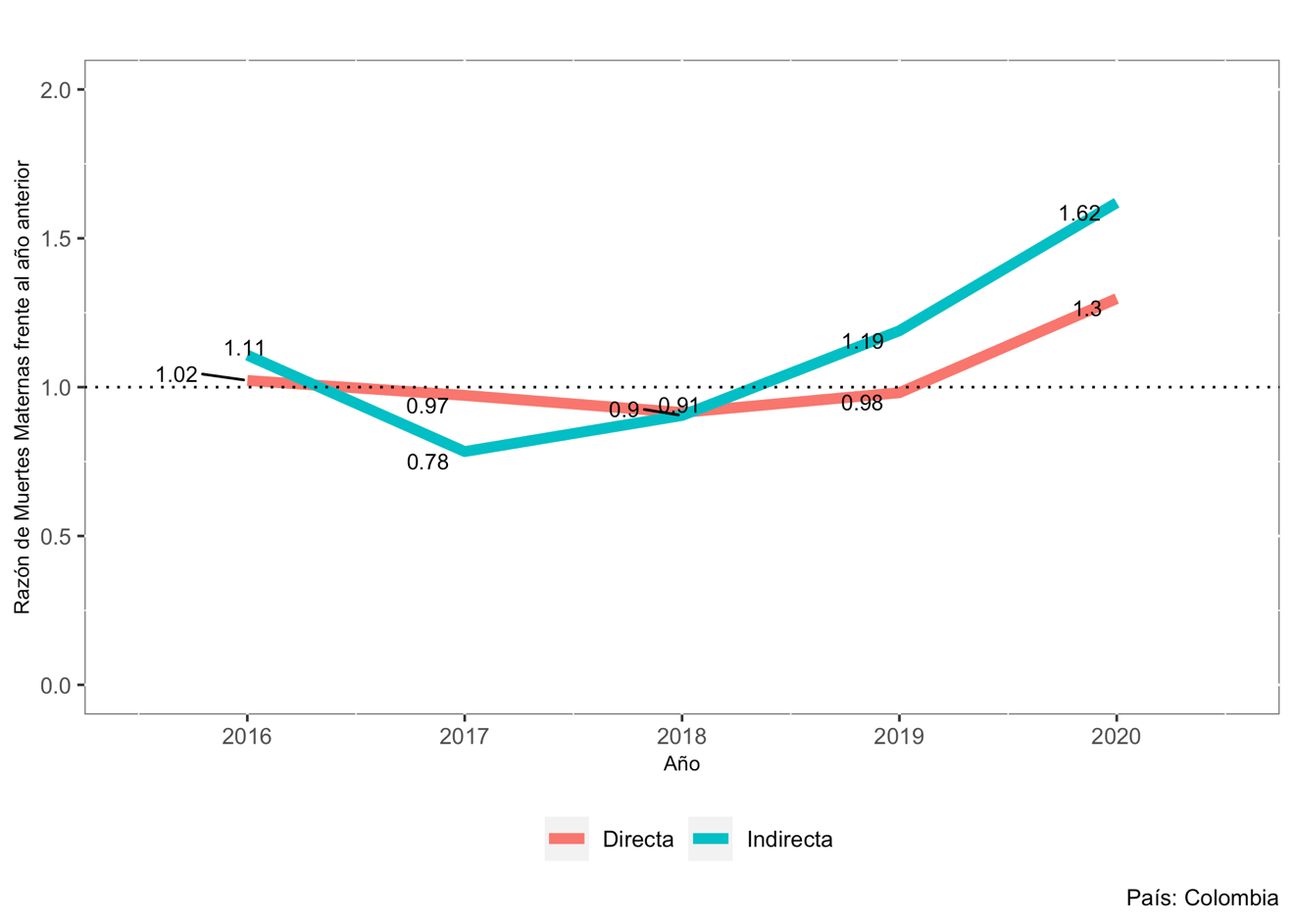


**Supplementary Figure 7: Maternal mortality by classification (direct/indirect). Ratio vs. previous year. 2015-2021. Country: Colombia**


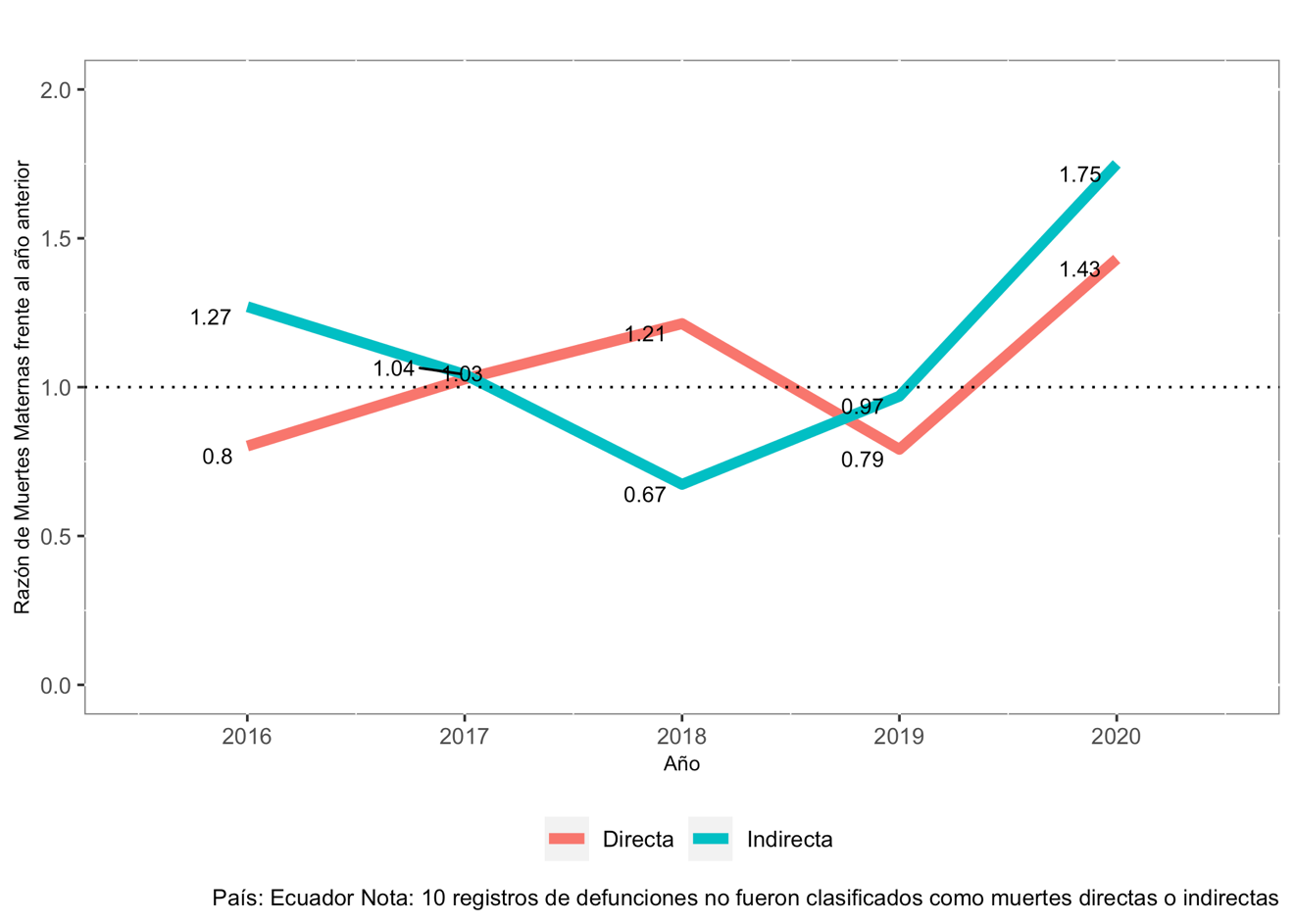


**Supplementary Figure 8: Maternal mortality by classification (direct/indirect). Ratio vs. previous year. 2015-2021. Country: Ecuador**

**NOTE: 10 registered deaths were not classified as direct or indirect**


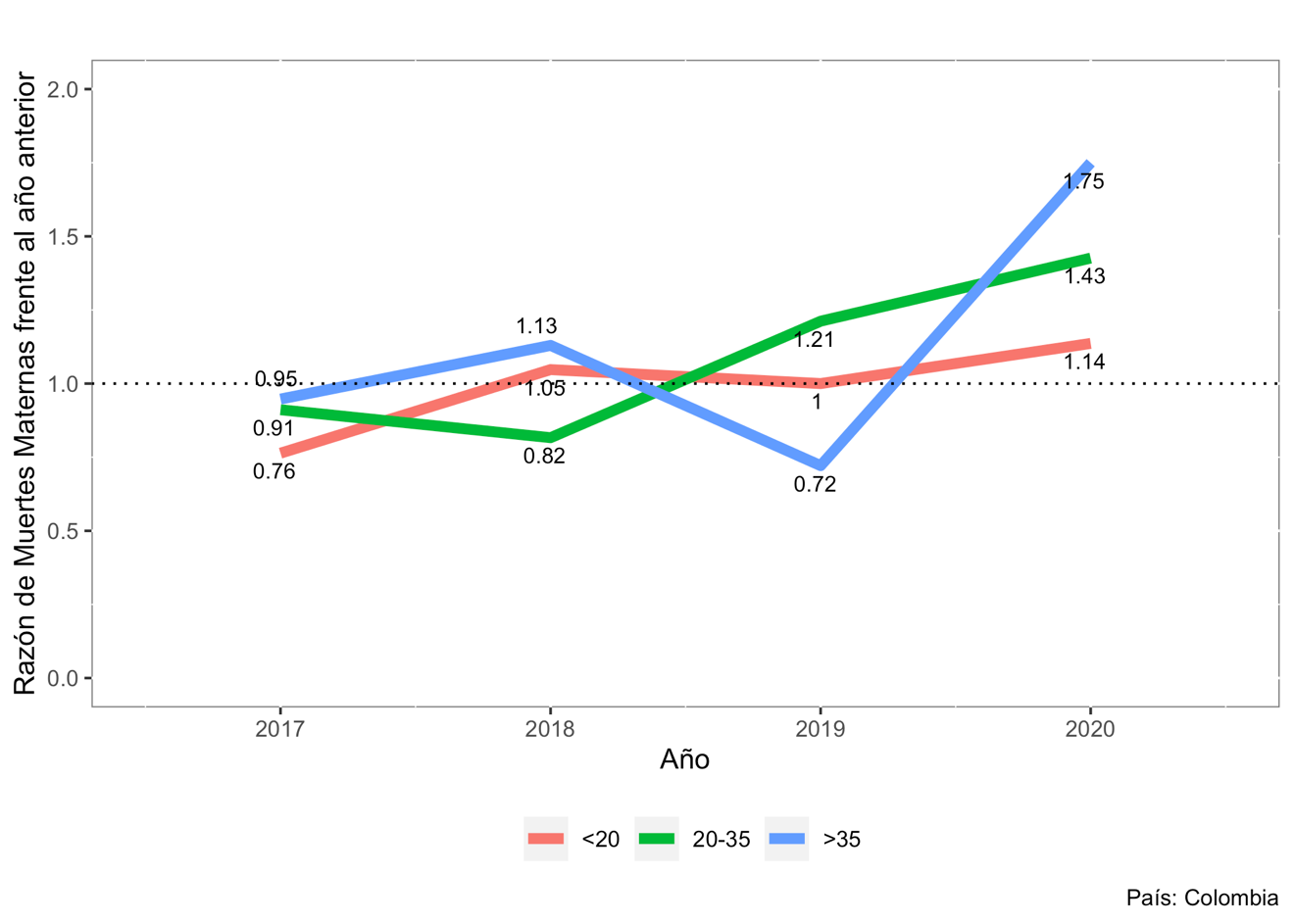


**Supplementary Figure 9: Maternal mortality by age group. Ratio vs. previous year. 2015-2021. Country: Colombia**


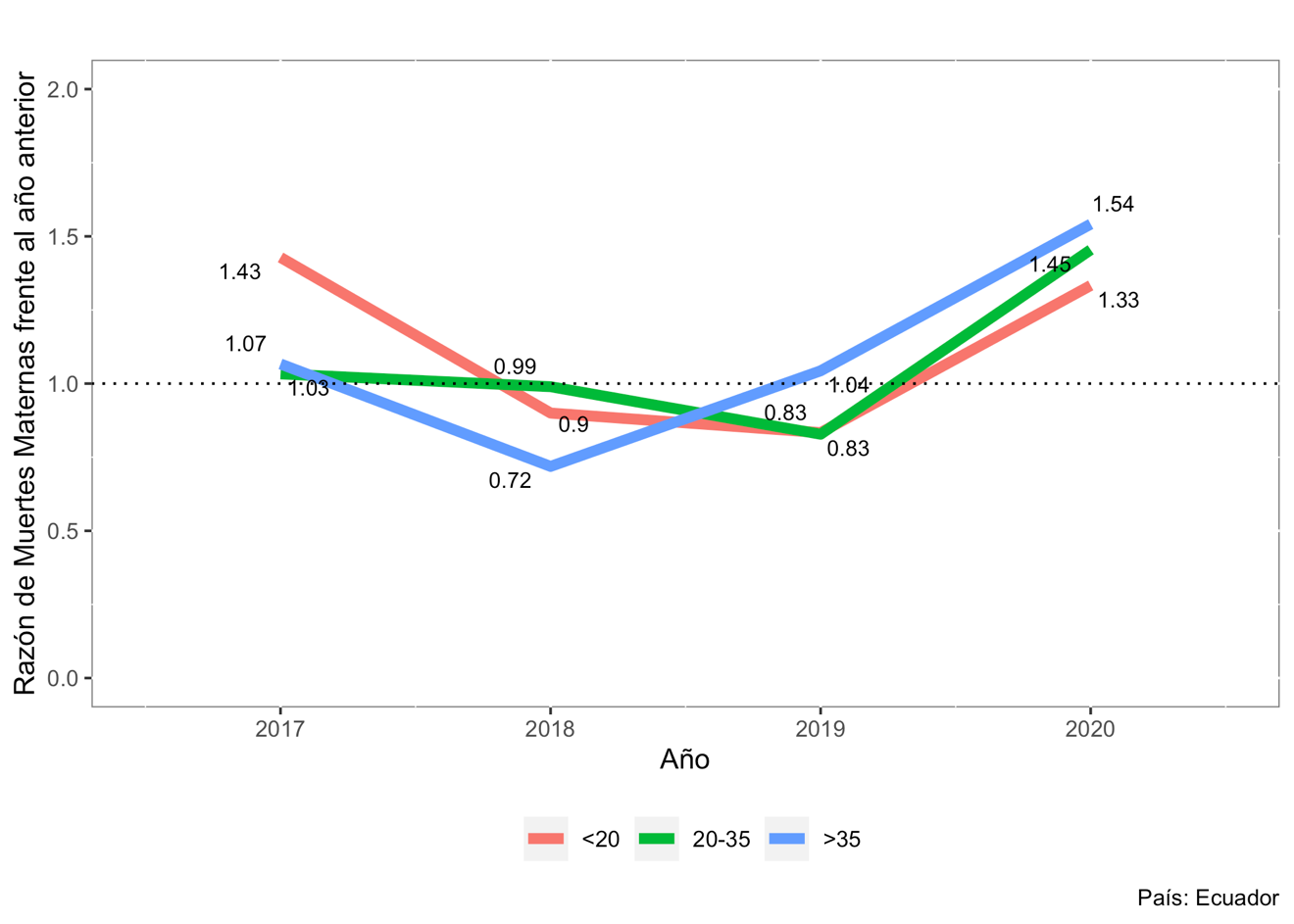


**Supplementary Figure 10: Maternal mortality by age group. Ratio vs. previous year. 2015-2021. Country: Country: Ecuador**


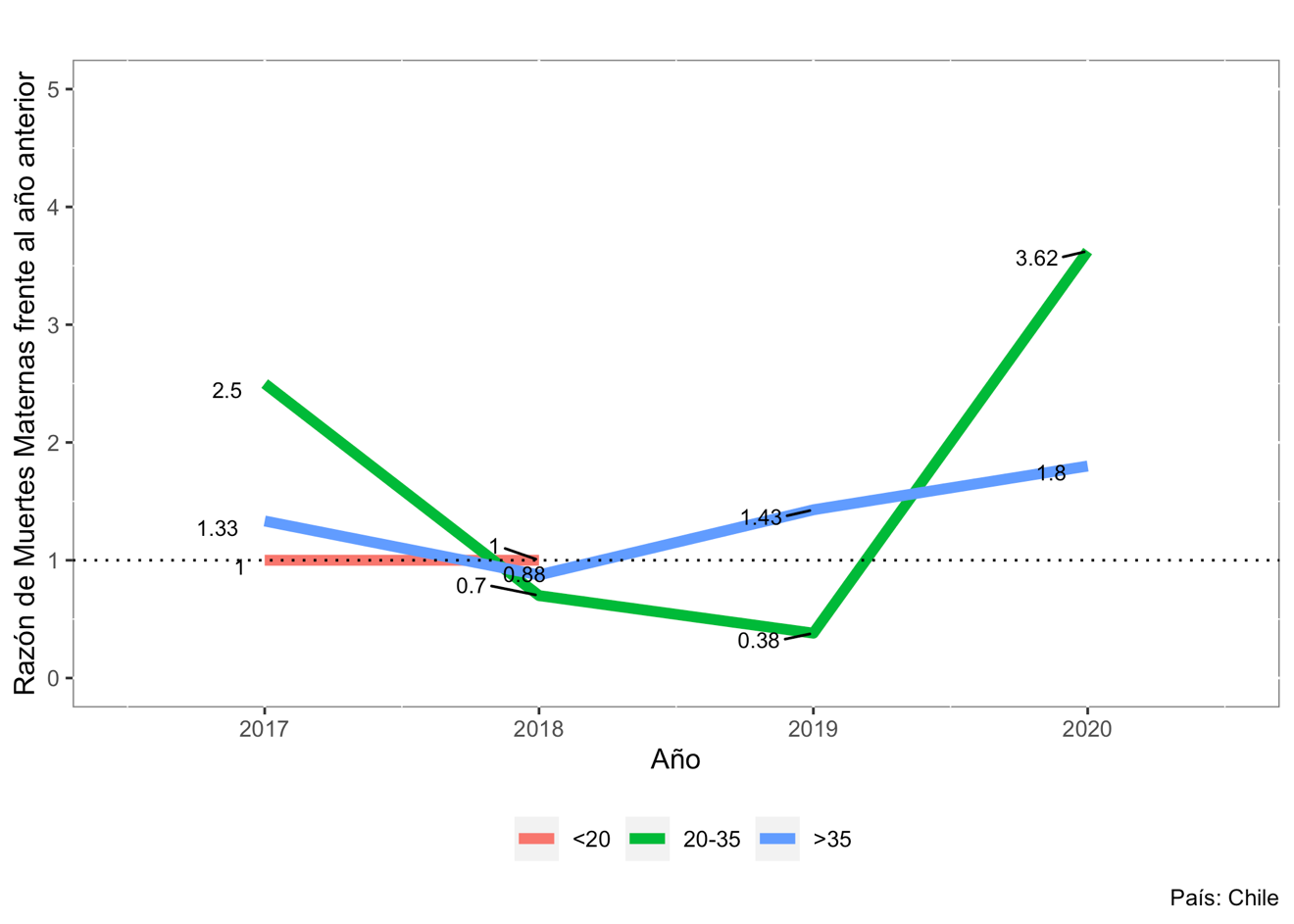
**Supplementary Figure 11: Maternal mortality by age group. Ratio vs. previous year. 2015-2021. Country: Chile**


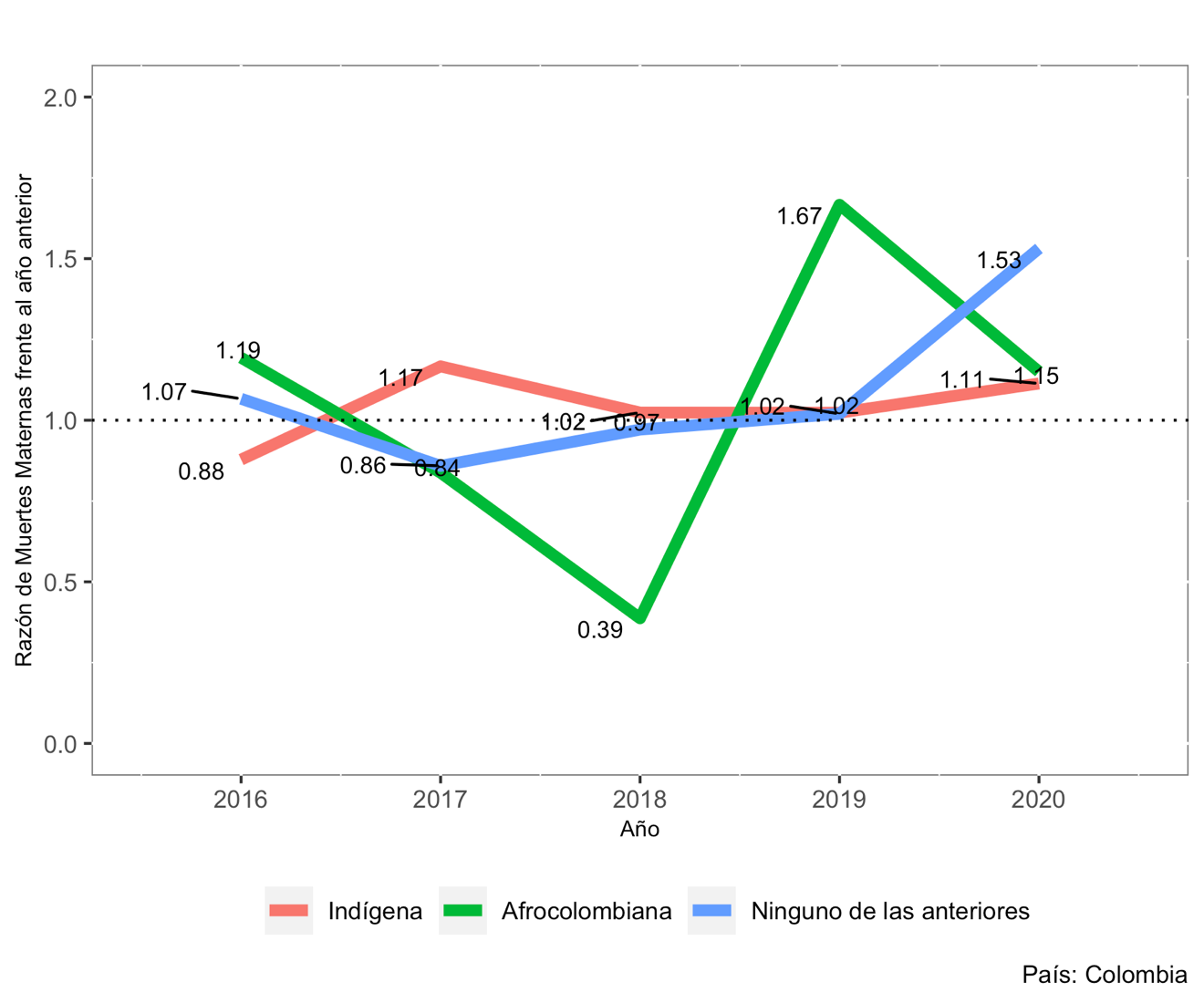


**Supplementary Figure 12: Maternal mortality by ethnicity and classification of death. Ratio vs. previous year. 2015-2021. Country: Colombia**

**RED = Indiginous; GREEN = Afro-Colombian; BLUE = Neither category**


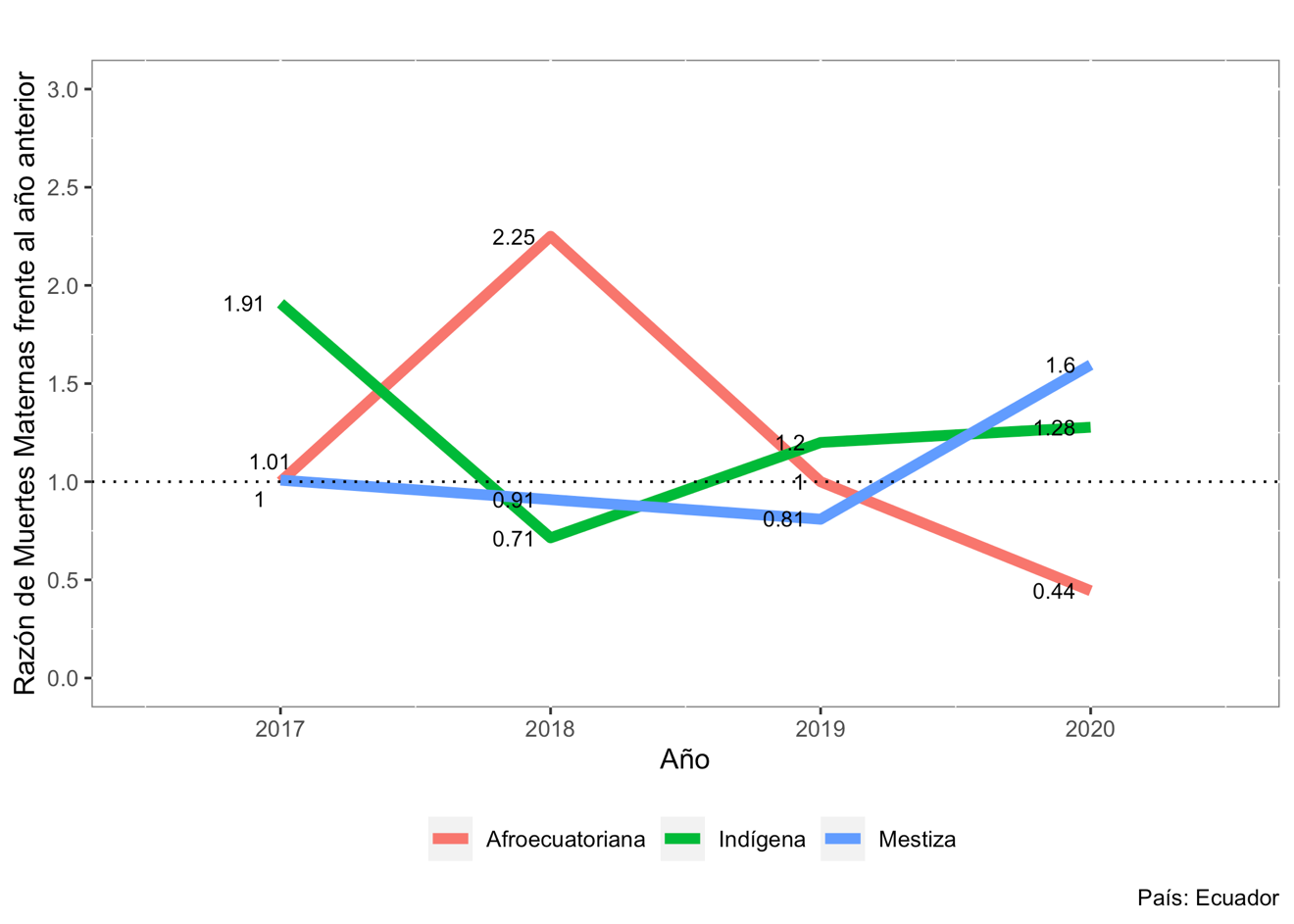


**Supplementary Figure 13: Maternal mortality by ethnicity and classification of death. Ratio vs. previous year. 2015-2021. Country: Ecuador**

**RED = Afro-ecuadorian; GREEN = Indigenous; Blue = Mixed race**

**
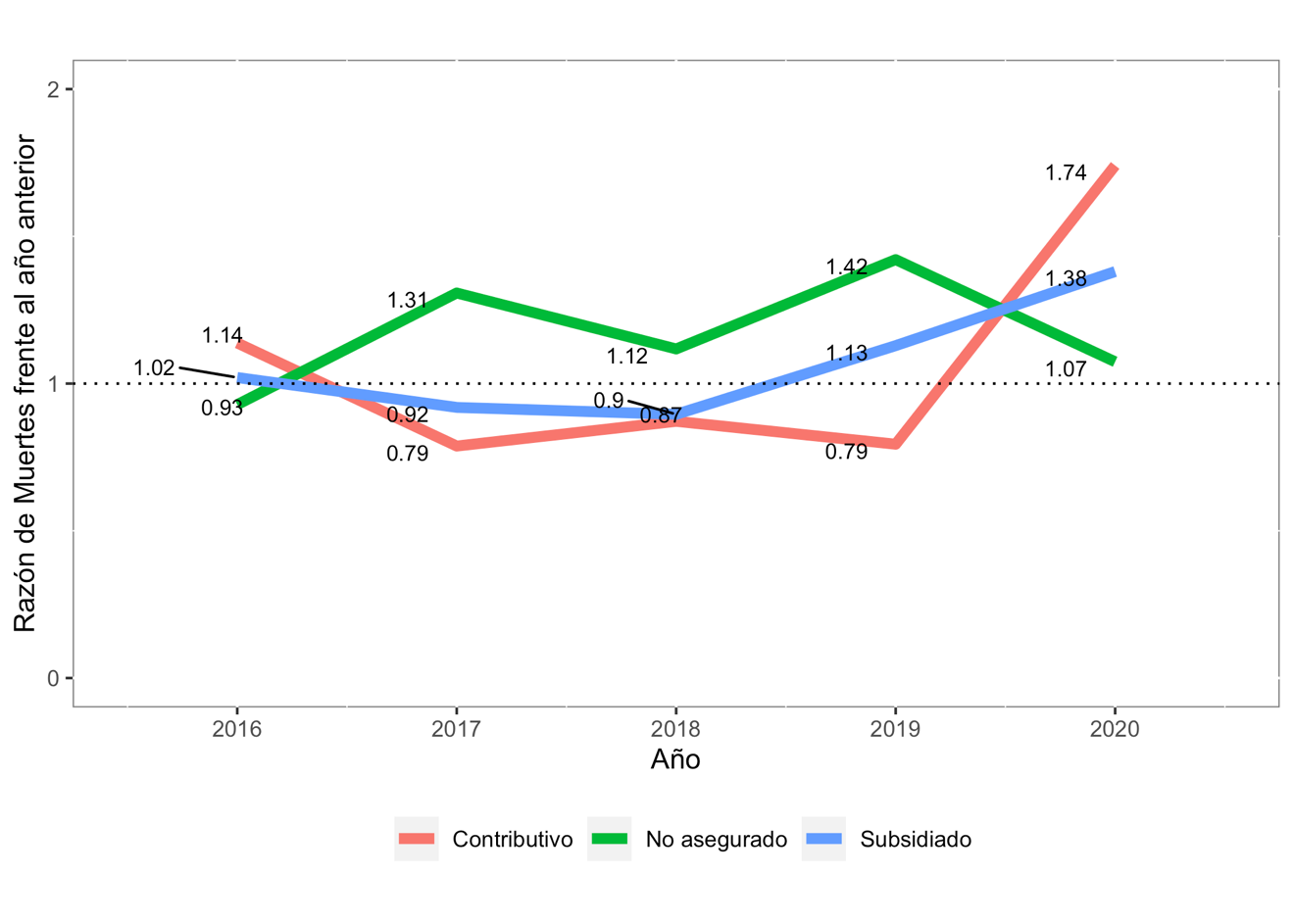
Supplementary Figure 14: Maternal mortality according to type of affiliation in the General Social Security Health System. Ratio vs. previous year. 2015-2021. Country: Colombia**

**RED = Insured; GREEN = Uninsured; BLUE = Subsidized**
